# A trade-off for covalent and intercalation binding modes: a case study for Copper (II) ions and singly modified DNA nucleoside

**DOI:** 10.1038/s41598-019-48935-2

**Published:** 2019-08-29

**Authors:** Jean-Marie Mouesca, Hania Ahouari, Sarath Chandra Dantu, Giuseppe Sicoli

**Affiliations:** 1 Grenoble University, CEA, CNRS, INAC–SyMMES, F–38000 Grenoble, France; 2 CNRS UMR 8516, Lille University, LASIR Institute–Bâtiment C5, Avenue Paul Langevin, F–59655 Villeneuve d’Ascq, France; 3 Department of Computer Science – Synthetic Biology Theme, Brunel University London, Kingston Lane, Uxbridge UB8 3PH, London, United Kingdom

**Keywords:** Physical sciences, Chemistry, Physical chemistry

## Abstract

Selective binding to nucleic acids and, more generally, to biopolymers, very often requires at a minimum the presence of specific functionalities and precise spatial arrangement. DNA can fold into defined 3D structures upon binding to metal centers and/or lanthanides. Binding efficiency can be boosted by modified nucleosides incorporated into DNA sequences. In this work the high selectivity of modified nucleosides towards copper (II) ions, when used in the monomeric form, is unexpectedly and drastically reduced upon being covalently attached to the DNA sequence in single-site scenario. Surprisingly, such selectivity is partially retained upon *non*-covalent *(i*.*e*. intercalation) mixture formed by native DNA duplex and a nucleoside in the monomeric form. Exploiting the electron spin properties of such different and rich binding mode scenarios, 1D/2D pulsed EPR experiments have been used and tailored to differentiate among the different modes. An unusual correlation of dispersion of hyperfine couplings and strength of the binding mode(s) is described.

## Introduction

Metal ions play important roles in many ribozyme-mediated examples of scission and ligation reactions but their precise role in those reactions in not yet fully understood^1–3^. The presence of an imidazole moiety in biological molecules has focused attention towards imidazole-modified nucleosides and the corresponding complex formed with copper (II) ions. Selected histamidine/histidine modified 2′-deoxyriboadenosines (histam^6^dA or hi^6^dA) have been found to be strong chelators for Cu(II) ions^4–6^. Their efficiency as chelators has been proven for the DNA cleavage reaction, revealing the intercalative ability of the complex Cu(II)/ligand^7^. Cu(II) ions have been also extensively used as cofactors for DNAzymes (deoxyzibozymes) for cleavage or ligation reactions, either on RNA or DNA strands. The highly densely modified DNAzyme sequence has been also tested and metal-independence has been demonstrated^8^. Covalently modified nucleoside has been used as paramagnetic probes (i.e. copper ions) for the determination of interspin distances^9^; intercalation of copper(II) ligand has been studied mainly by CW EPR for elucidating the structure of DNA-fiber^10^. To make this scenario more challenging, ligation and cleavage reactions often make use of “metal soup”^11^ as cofactor or metal-lanthanide binary cofactor^12^, and the role of different cofactors in the catalytic process still remains unknown. In order to localize the binding region(s) over DNA or RNA sequences, selective chemical modifications have been tested^13^; such an approach has revealed itself to be tedious and does not solve the major issue concerning the identification of binding sites of metal and lanthanide cofactors with respect to the nucleotide structure, with a particular focus on single or multiple sites. Taking into account that such systems very often contain paramagnetic species^14^, NMR spectroscopy is not the most suitable method for the analysis of the structural features of these complexes. Absorption^15^ and fluorescence spectral studies^16^ are more conventional methods to analyze these complexes, but they only provide a global view of the system under investigation and very often do not provide information on the nature of the nucleus/nuclei forming the coordination sphere of the metal center. Electron paramagnetic resonance (EPR), known also as electron spin resonance (ESR), offers a plethora of experiments for detection of short- and long-range interactions. Hyperfine spectroscopy is leading the structural analysis of the catalytic cores of several proteins, especially those containing iron-sulfur clusters as initiators of radical species^17–19^. Among these, the more conventional pulsed EPR technique, HYperfine Sublevel COrRElation spectroscopy (HYSCORE) and the emerging technique ELDOR-Detected NMR (EDNMR) are methods of choice for evaluating interactions of the paramagnetic species and its surrounding nuclei. In particular, ELectron-electron DOuble Resonance (ELDOR)-detected NMR (EDNMR) is gaining popularity due to the latest developments in wave-generator and multi-frequency approaches^20,21^. This sequence was first developed in Schweiger’s laboratory^22^, and uses selective microwave pulses which simultaneously pump EPR and NMR transitions of the spin manifold, so called spin forbidden transitions, where both the electron and nuclear spins change their projection direction. As compared to conventional Electron Nuclear DOuble Resonance (ENDOR) techniques it has a much higher sensitivity and does not exhibit nucleus- or pulse-dependent spectral artifacts^23^. Historically, the widespread adoption of EDNMR has been hampered by what is termed the “central blind spot”. The limited resolution is mitigated by performing the EDNMR experiment at higher magnetic fields (>9 GHz) as demonstrated by a number of recent studies^24^. Furthermore, the multi-frequency approach can combine the advantages of low field optimized experiments (HYSCORE) with those at higher field (EDNMR)^25,26^. EPR/ESR hyperfine spectroscopic methodologies have been successfully combined with the analysis of metalloenzymes that exhibit high selectivity and specificity for catalytic processes involving radical species^27^. The restricted alphabet of nucleic acids compared to amino acids for proteins is often associated with lower selectivity and specificity, and even for ribozymes, the main role for the catalytic process is extensively recognized with the protein moiety associated with metal cofactors. However, a minimalistic approach for testing binding modes is related to the analysis of emerging and promising structures such as for DNAzymes (deoxyribozymes) where the role of the metal cofactor remains to be understood, as well as that of lanthanide cofactor^28^. The discovery of new structures, very often associated with a serendipitous approach^29^, is severely lacking with respect to an accurate recognition and description of (*i*) binding mode(s) and (*ii*) binding site(s) from the perspective of the DNA structure^30^, which may provide a lower differentiation than that available in the catalytic core of proteins.

In this work two main aspects have been taken into account concerning the incorporation of a copper(II) chelator into DNA sequences. First, a short overview of two different nucleosides containing an imidazole moiety is reported; second, the binding modes of the modified nucleoside have been analyzed by two different scenarios: (*i*) covalent attachment to the DNA sequence, and (*ii*) non-covalent (intercalation) mixture of nucleoside and Cu^2+^ ions with the native DNA sequence. The two structures differ in the connection of the imidazole ring to the nucleoside: in one case an ethyl-bridge has been used and in the second structure a urea-ethyl-bridge has been used, connecting the nucleoside moiety to the imidazole moiety. A crowded scenario of binding modes (and binding sites) involves: (*a*) copper(II) free in solution, (*b*) the monomeric form of the nucleoside combined with Cu^2+^, (*c*) native DNA duplex combined with Cu^2+^, (*d*) covalently modified DNA sequence with one singly modified nucleotide combined with Cu^2+^, (*e*) native (unmodified) DNA sequence with non-covalently attached (intercalation-type) monomeric nucleoside, combined with Cu^2+^. Those five different binding modes have been correlated to hyperfine spectroscopy (interactions electron-nuclei) to implement the core of such a fast and straightforward protocol, in order to confirm (or discard) the formation of a complex and to provide preliminary information on the nature of the nuclei that comprise the coordination sphere.

## Results

### Binding efficiency retained

**Figure 1:**
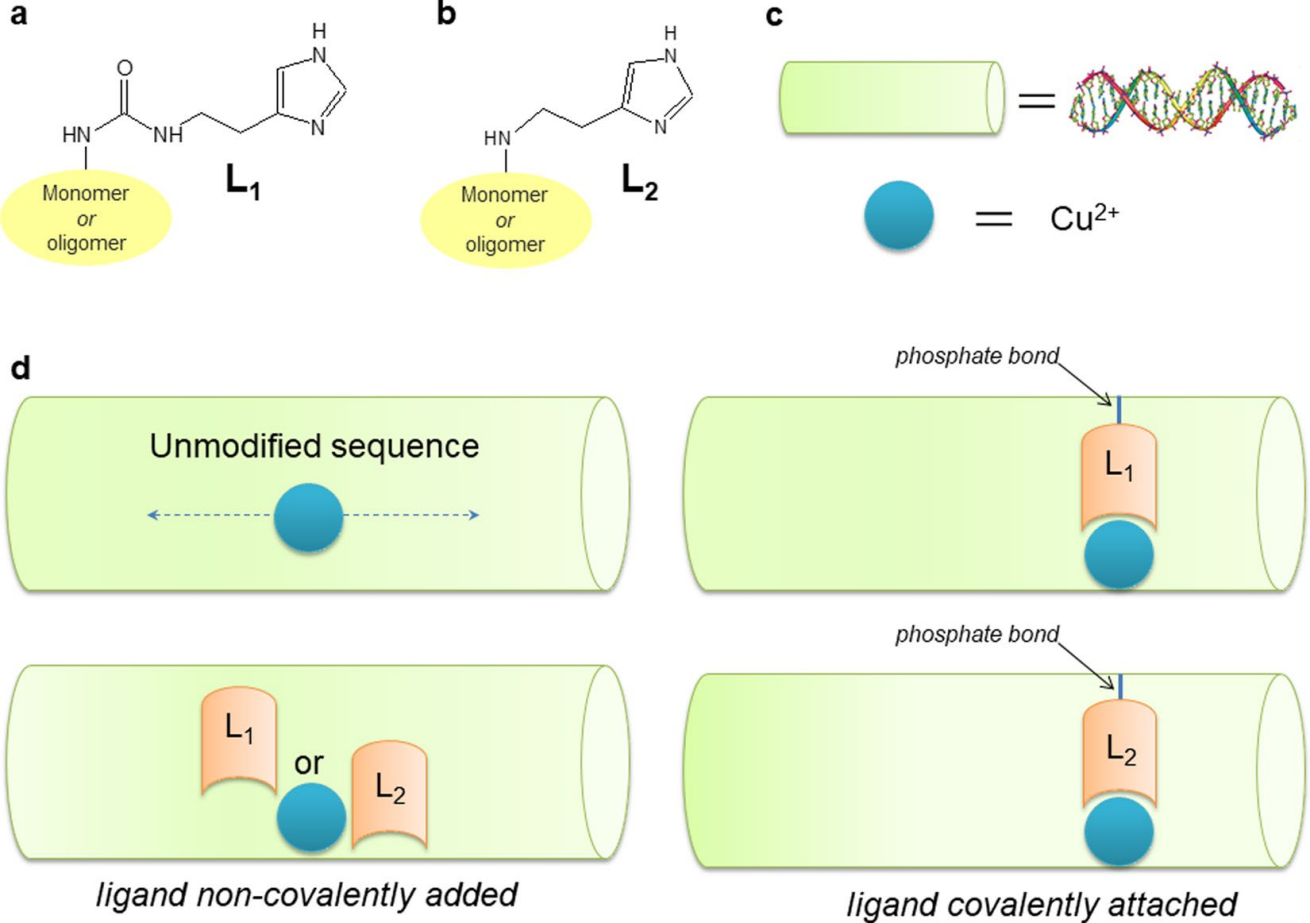
(**a**,**b**) Schematic view of attached modes for the imidazole moiety to the nucleoside. (**c**) Simplified schematic view of duplex and paramagnetic center (Cu^2+^) involved in this study. (**d**) Free copper(II) in solution added to the unmodified duplex, L_1_ and L_2_ covalently attached to the duplex and L_1_ and L_2_ used as intercalating agents.

**Figure 2:**
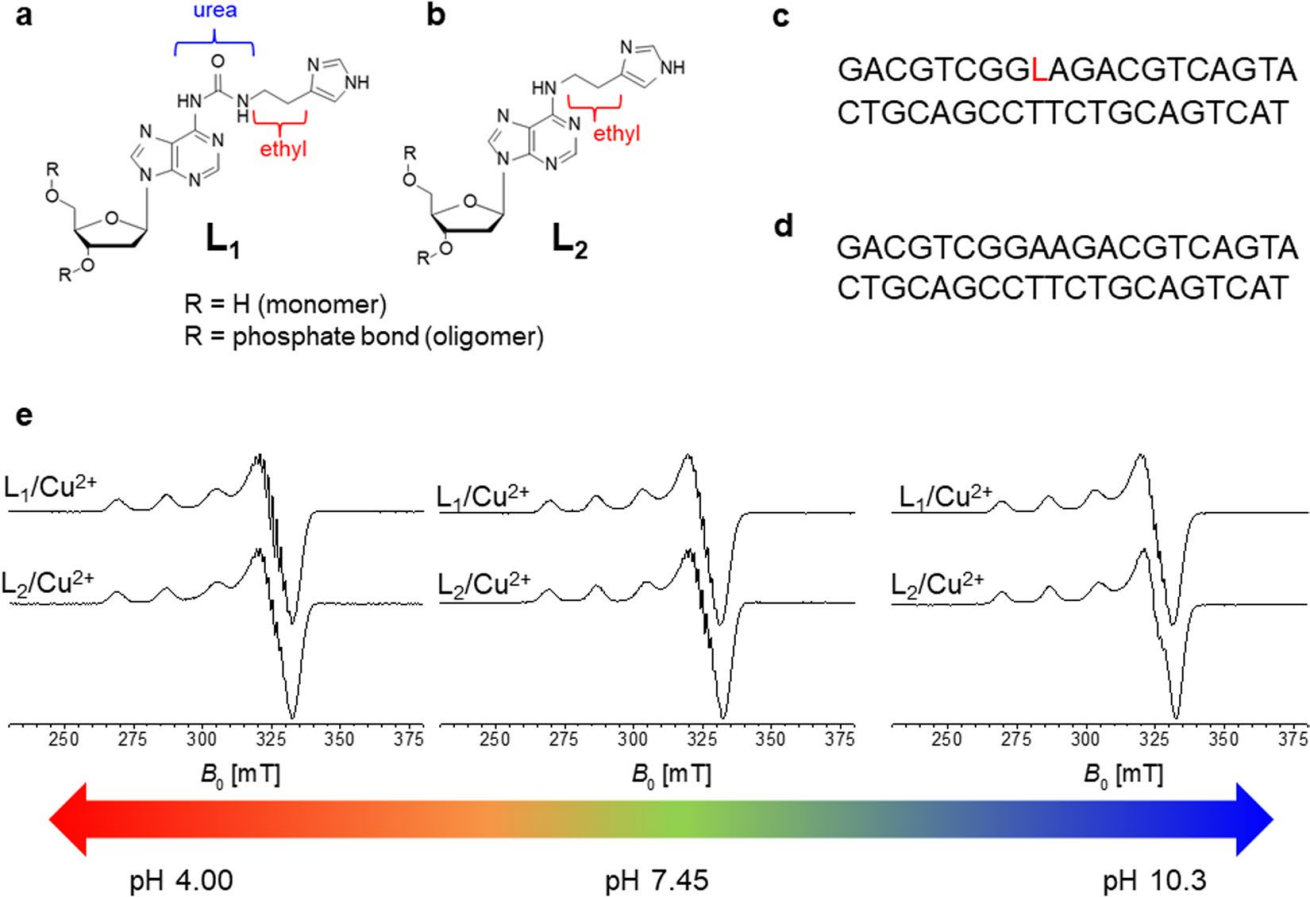
(**a**) Detailed structure of the old ligand L_1_, connecting the imidazole moiety and the nucleoside *via* an ‘ethyl-urea-bridge’. (**b**) Detailed structure of the ligand L_2_, connecting the imidazole moiety and the nucleoside *via* an ‘ethyl-bridge’. (**c**) Covalently modified DNA sequence. (**d**) Native DNA sequence formed by 20 nucleotides. (**e**) CW EPR spectra for the monomeric form of ligands L_1_ and L_2_, recorded at three different pH values (4.00, 7.45 and 10.3).

**Figure 3:**
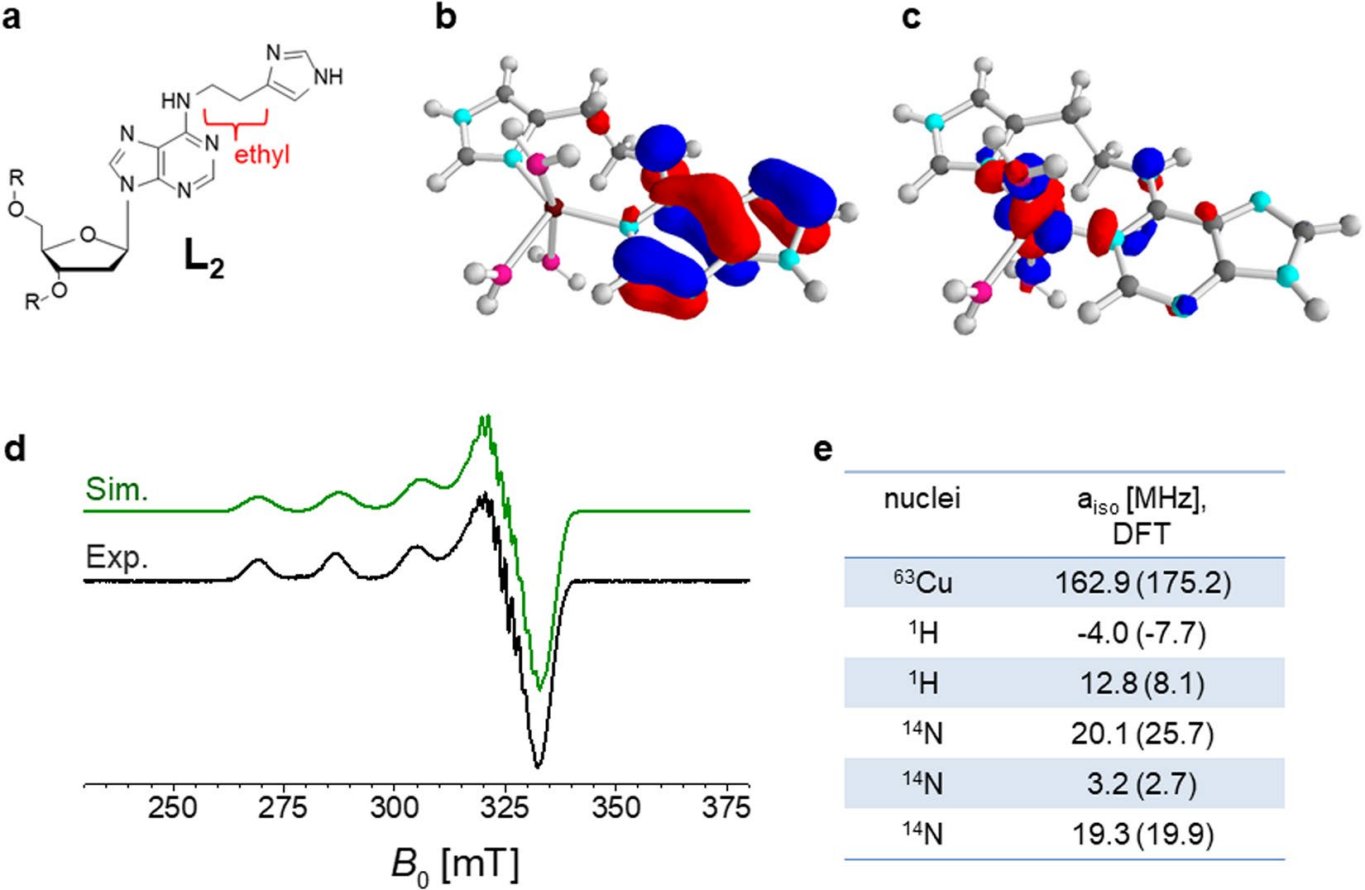
(**a**) Structure of ligand L_2_ (ethyl-bridge is highlighted for clarity). (**b**,**c**) Optimized structure obtained by DFT calculations at pH 7.45 (hyperfine couplings are computed for all nuclei forming the structure); *left*: plot of the LUMO orbital with iso-density value ±0.05 a.u.; *right*: plot of the SOMO orbital with iso-density value ±0.05 a.u. (**d**) Simulated EPR spectra using DFT calculated hyperfine values (green line) and comparison with the experimental spectrum (black line). (**e**) Table summarizing the DFT hyperfine couplings for the different nuclei (six) used also for fitting procedure (with hyperfine coupling higher than 2.5 MHz). In bracket the isotropic values of hyperfine couplings used for the fit of experimental spectra (Easyspin software version 5.2.21)^29^.

The analysis of different binding modes focused on two ligands, shown in Fig. 1a,b. The overall study involved the insertion of the monomeric form of the imidazole moiety into DNA duplexes (indicated in Fig. 1c), both as covalently attached and in a non-covalent mixture, in order to estimate the different binding modes for Cu^2+^. In Fig. 2a,b details of ligands L_1_ and L_2_ are depicted. An ethyl bridge, introduced into the structure L_2_, has replaced the ethyl-urea bridge between the nucleoside and the imidazole fragment that characterizes the modified nucleoside L_1_. Figure 2c,d illustrate the DNA sequences containing one modified nucleotide and the native sequence, respectively. The L_2_ monomer was analyzed by CW and pulsed EPR spectroscopy. As for the L_1_ ligand, the binding mode of copper(II) was tuned by different pH values and different ligand/copper ions ratios (Supplementary Information S1 shows how the EPR/ESR spectra are affected by pH changes in the mixture 1:2, Cu^2+^/L). In this study we aim to go beyond the impact of the pH values on the coordination of copper ion(s); testing different Cu^2+^/ligand ratios, we can guarantee a full coordination at a ratio 5:1 (ligand/Cu^2+^), as shown in Supplementary Information S2 and S3. Furthermore, operating at this ratio, a very similar trend has been observed for the two different ligands, as reported in Fig. 2e; the subsequent analysis has been then focused on the solution at pH 7.45 and with a ratio 5:1 (ligand/Cu^2+^). Such reduced influence of pH within the range 4.00–10.3 increases the amount of species with complete copper ion coordination sphere, ensuring a negligible effect on species distributions^4–6^. Those distributions involve the equilibrium between bound and free states of the copper ions, as well as monomeric and dimeric forms of the formed adducts. The analysis of monomeric L_1_ and L_2_ with Cu^2+^ has been combined with extensive DFT computational studies. The hyperfine couplings obtained by those calculations have been used as starting values for the simulation of spectral features. Indeed, in Fig. 3b,c the LUMO and SOMO models, respectively, derived by DFT calculations are depicted. The LUMO model would be the best molecular representation of the distribution of the hyperfine coupling constants, while the SOMO model confirms the occupied orbital for the unpaired electron of this paramagnetic complex. Furthermore, in Fig. 3d the comparison between experimental and simulated CW EPR spectrum is reported for the complex Cu^2+^/L_2_ at pH 7.45. In Fig. 3e the isotropic hyperfine couplings for this complex are reported, both from DFT computations and EasySpin fitting procedures (in brackets)^31^.

Slight discrepancies exist between experimental and simulated spectra, caused by the presence of minor species in the equilibrium of bound and free states. The comparison of the six complexes (L_1_ and L_2_ ligands at six different pH values) is provided in the Supplementary Information S4. The full comparison of the monomeric forms of the two ligands demonstrates the retained efficiency of the structure L_2_, where the binding properties are analogous to those of L_1_, even in the absence of the urea bridge. For the simulation of the monomeric form (i.e. at pH 7.45, for L_2_) six nuclei were used: ^63^Cu, two ^1^H, two strongly coupled ^14^N (hyperfine coupling around 20 MHz for both atoms) and one weakly coupled ^14^N, placed in the so-called remote position of the imidazole moiety. The agreement between the experimental of simulated spectrum prompt us to extend the comparison of the DFT computed hyperfine couplings with the experimental spectra of L_1_ and L_2_ ligands. Tables composing the ensemble (Supporting Information S4) support the slight differences detected for the monomeric form of the ligands. A very similar trend is observed for L_1_ and L_2_: the ethyl-bridge does not perturb the formation of the complexes. Supporting Information S5 summarizes the six structures derived by DFT computational analysis, in the same manner as that single example depicted in Fig. 3b,c. Details about DFT calculations and optimizations are provide in the Methods section.

Among the hydrogen atoms coupled to the copper paramagnetic center, strongly coupled exchangeable protons have not been detected (Supporting Information S6). This supports the scenario where several heteronuclear couplings (*i*.*e*., ^14^N) are strongly correlated to the copper paramagnetic center.

### Echo field sweep as a first diagnostic differentiation

**Figure 4:**
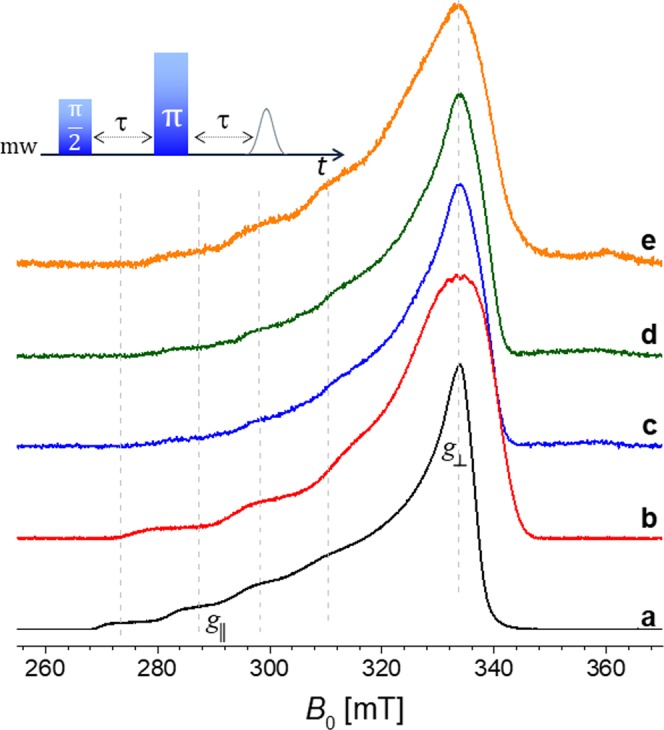
Echo-FS spectra recorded at 9.72 GHz for the following samples: (**a**) CuCl_2_·2H_2_O in H_2_O. (**b**) Monomeric L_2_ ligand (‘ethyl-bridge’) with CuCl_2_·2H_2_O in a cacodylated buffer solution (pH 7.45). (**c**) Native (unmodified) DNA sequence with CuCl_2_·2H_2_O in a cacodylated buffer solution (pH 7.45), using an excess 5:1. (**d**) Singly modified DNA sequence (L_2_ ligand covalently attached) with CuCl_2_·2H_2_O in a cacodylated buffer solution (pH 7.45), using an excess 5:1. (**e**) Monomeric L_2_ ligand mixed with native DNA sequence (NON-covalently attached) with CuCl_2_·2H_2_O in a cacodylated buffer solution (pH 7.45), using an excess 5:1.

The analysis of different echo field sweep spectra recorded at low field (9 GHz) provides a first overview on the strength of the copper binding. The five spectra in Fig. 4 show a typically axial g-tensor for the copper center; slight deviation from axial to rhombic are shown in Supporting Information S4 (Tables summarizing the parameters used for EPR spectra simulations). The broadening of the perpendicular component of the tensor (g⊥) is observed especially in Fig. 4b,e (red and orange lines, respectively). Compared to free copper in solution (Fig. 4a, black line), the complex formed by monomer L_2_ and copper ions is expected to provide such broadening. In case of the orange line (Fig. 4e), it concerns the mixture formed by copper ions, native DNA duplex and monomeric L_2_, interacting in a non-covalent manner with respect to the DNA sequence (intercalation). Such a three-component complex generates a small broadening compared to the monomeric L_2_/Cu^2+^ complex, but higher than that generated by duplexes, both native or singly-modified (Fig. 4c,d, respectively). Surprisingly, the covalently attached ligand L_2_ does not guarantee a strong coordination, even if the modified DNA sequence is used with a molar excess (5:1).

### HYSCORE experiments

**Figure 5:**
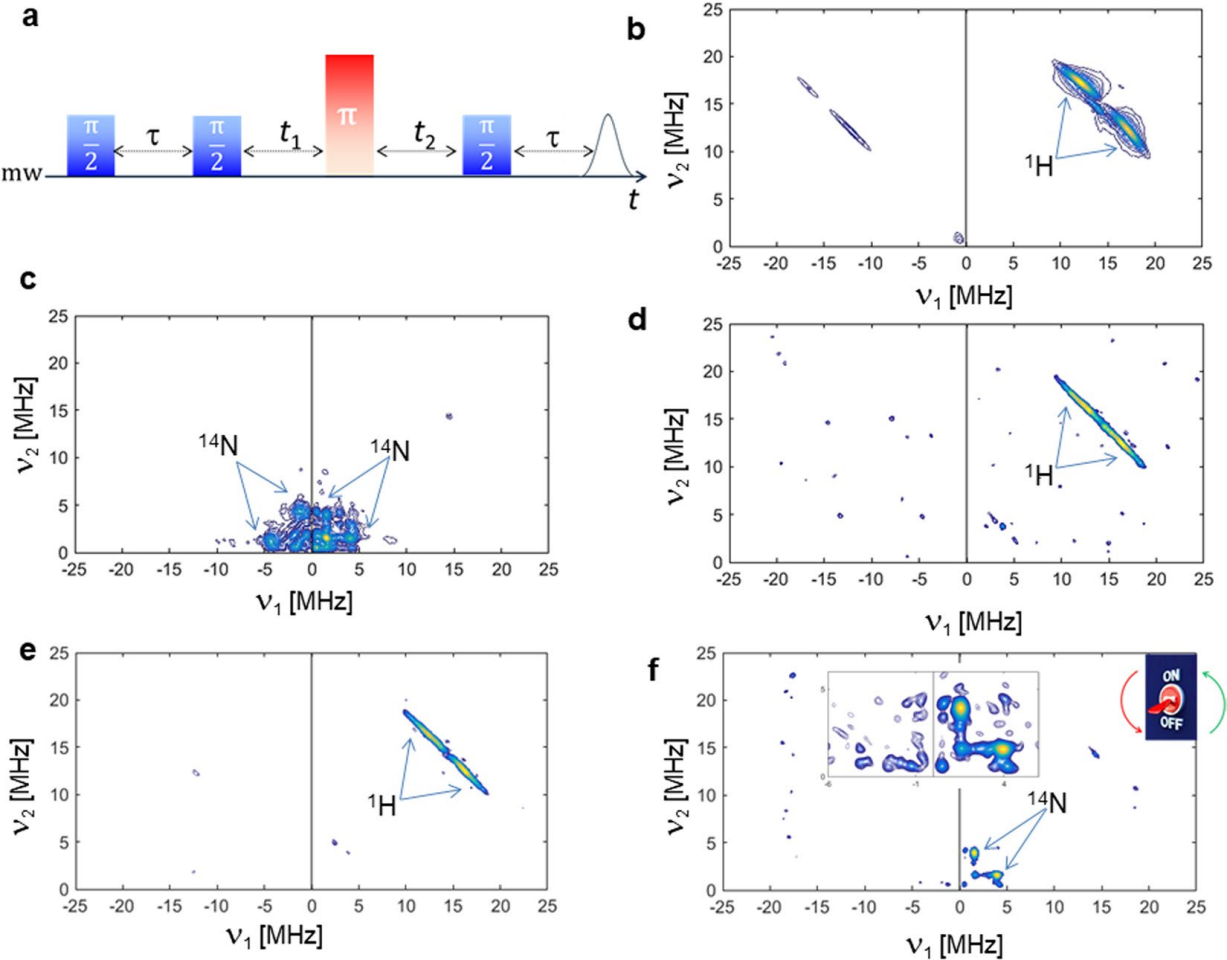
(**a**) 2D HYSCORE pulse sequence; the experiments for the 5 samples indicated in **b**–**f** are recorded at 20 K. (**b**) CuCl_2_·2H_2_O in a cacodylated buffer solution (pH 7.45). (**c**) Monomeric L_2_ and Cu^2+^. (**d**) Native DNA duplex and Cu^2+^. (**e**) Covalently modified DNA containing one L_2_ ligand and Cu^2+^. (**f**) Three components mixture containing native DNA sequence, monomeric L_2_ (non-covalently added) and Cu^2+^.

Five solutions at pH 7.45 were then analyzed by 2D HYSCORE experiments for the detection of weak hyperfine couplings (<10 MHz). Two main regions can be identified for the hyperfine couplings, involving ^14^N and ^1^H nuclei. The 1:2 ratios (Cu^2+^/L_1_) also indicated the relative amounts of those nuclei with respect to three different pH values (Supporting Information S7, S8 and S9). Focusing on the solution at pH 7.45, five different solutions were studied. In Fig. 5a the HYSCORE sequence is depicted. Figure 5b shows the free Cu^2+^ in solution, without any monomeric ligand or DNA sequence. The main hyperfine coupling detected for this mixture is related to ^1^H, with a Larmor frequency centered at 14 MHz and a hyperfine coupling of 5 MHz. A completely different scenario is offered by the monomeric L_2_ ligand and Cu^2+^ at pH 7.45 (Fig. 5c). The main hyperfine coupling observed is that of the remote ^14^N (with a coupling of 2.7 MHz); the dynamics of the complex and the distribution of radical species in this sample cover the entire region of single and double transitions (besides the combination frequencies), rendering this crowed region quite difficult to disentangle, but confirming the binding proposed by CW experiments. The spectra depicted in Fig. 5d,e show ^1^H to be the predominant nucleus within the hyperfine pattern; 5*d* is the native duplex added to Cu^2+^ solution at pH 7.45 while the 5*e* is the modified duplex containing one L_2_ ligand (covalently attached) added to Cu^2+^ solution at pH 7.45. No significant differences were detected between these two samples. The presence of one singly modified nucleotide with a 20-mer DNA sequence does not guarantee the same binding exhibited by the monomeric L_2_ ligand. Such a loss of binding strength is unexpected, if compared with the monomeric form of the ligand. An attempt to recover the coupling with ^14^N centers was made by preparing a non-covalent mixture (intercalation of L_2_). The ligand L_2_ was indeed added (still with a ratio 5:1 with respect to the Cu^2+^ ion) to the native DNA sequence. The weak coupling is then detected in the order of 3.0 MHz.

### ELDOR-Detected NMR (EDNMR) experiments

**Figure 6:**
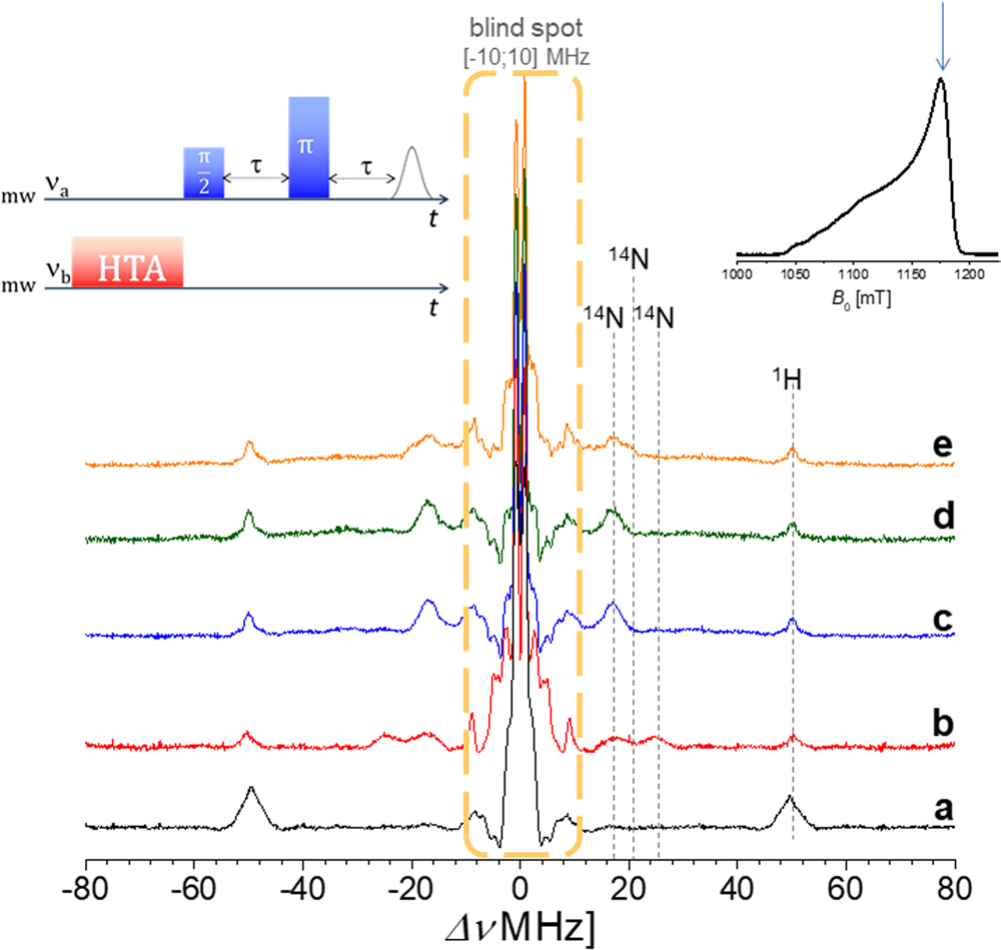
ELDOR-detected NMR spectra recorded at Q-band frequencies (*ν*_0_ = 34.025 GHz) and 20 K. The magnetic field was selected to be at the position of the maximum of the echo-FS spectra. The EDNMR are recorded on the following samples: (**a**) CuCl_2_·2H_2_O in H_2_O. (**b**) Monomeric L_2_ ligand (‘ethyl-bridge’) with CuCl_2_·2H_2_O in a cacodylated buffer solution (pH 7.45). (**c**) Native (unmodified) DNA sequence with CuCl_2_·2H_2_O in a cacodylated buffer solution (pH 7.45), using an excess 5:1. (**d**) Singly modified DNA sequence (L_2_ ligand covalently attached) with CuCl_2_·2H_2_O in a cacodylated buffer solution (pH 7.45), using an excess 5:1. (**e**) Monomeric L_2_ ligand mixed with native DNA sequence (NON-covalently attached) with CuCl_2_·2H_2_O in a cacodylated buffer solution (pH 7.45), using an excess 5:1. ^1^H signals are centered around |±*ν*_1H_| = 51.4 MHz, while the ^14^N signals are to the different values of |*ν*_1H_ ± A/2|. The total amount of ^14^N signals implies the contribution of the native duplex and the L_2_ ligand, both in the covalent and non-covalent binding mode. In all cases the strength of the detection pulses was selected to yield a maximum echo intensity.

**Figure 7:**
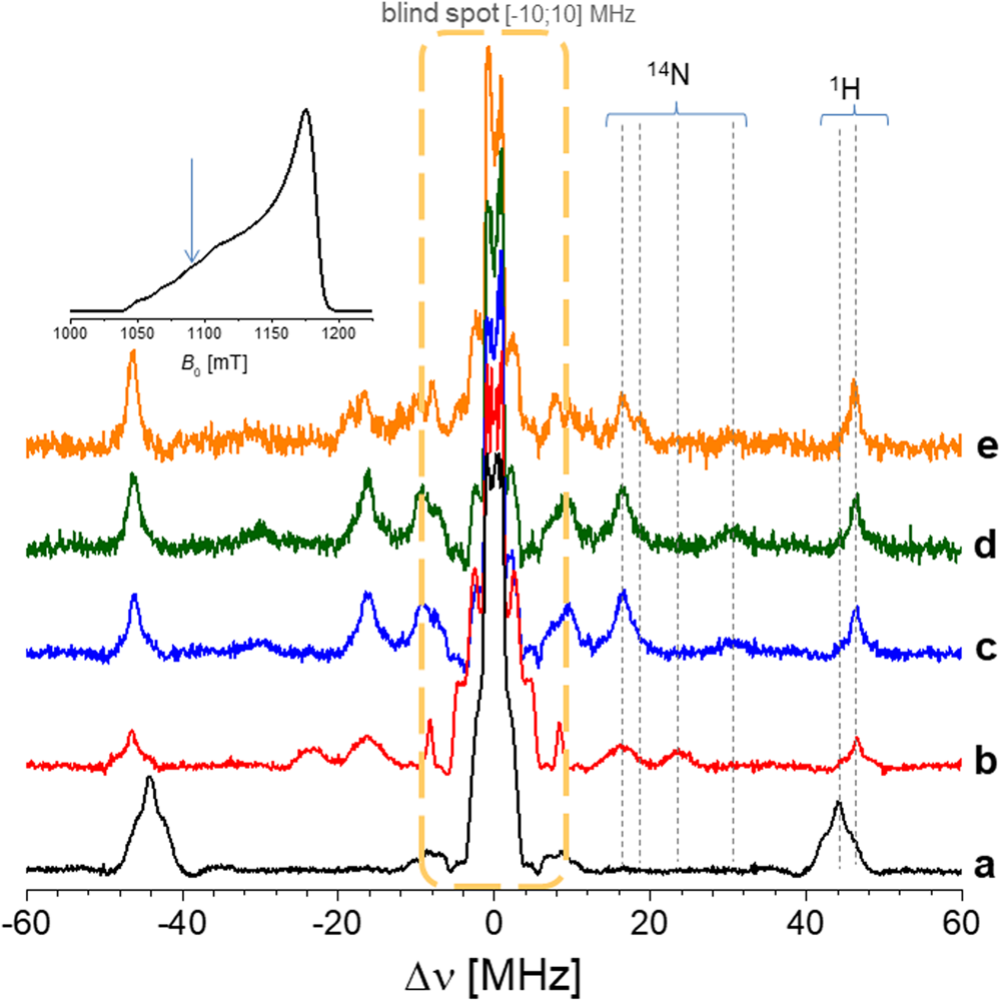
ELDOR-detected NMR spectra recorded at Q-band frequencies (*ν*_0_ = 34.025 GHz) and 20 K. The magnetic field was selected to be at the position indicated on the echo-FS spectra (*top-left*). Other experimental details as for Fig. 6.

The observed B values for EDNMR experiments have been selected on the echo-field sweep spectra recorded at 34 GHz (Supporting Information S10). The differences among different samples are here less pronounced, due to the additional broadening generated at higher field/higher frequency. For the EDNMR recorded at 34 GHz, a crucial role is played by the blind spot generated by the low frequency couplings. It was then necessary to optimize the width of the central blind spot; making it narrow enough so as not to obscure signals from any low-*γ* nuclei present while at the same time using an ELDOR pulse of sufficient power to excite the formally forbidden electron and nuclear transitions. Consequently, Q-band EDNMR is always a compromise between the signal-to-noise ratio and the width of the central blind spot. For our Q-band studies, the most convenient setup consisted of a 9000–400–800 ns pulse scheme (pulse sequence on the top-left of the figure). Thus, Fig. 6 shows a series of Q-band EDNMR spectra of different Cu^2+^ complexes; namely the hexa-aqua complex ([Cu(H_2_O)_6_]^2+^) (Fig. 6a), the mixture of Cu^2+^ with L_2_ ligand (Fig. 6b). In addition to the signal corresponding to the ^1^H nuclei (49.7 MHz), two broad signals corresponding to strongly coupled ^14^N were observed at 17.2 and 24.7 MHz, respectively. Signals within the blind spot regions (Supplementary Information S11) and related to the setup ambiguity have not completely assigned. The signals at 2.52 and 4.55 MHz have been identified as single-quanta (sq) EDNMR transitions of the ^14^N nucleus (*ν* ^14^N = 3.7 MHz, *I* = 1), as they are centered around the *ν*_14N_ Larmor frequency and split by 1.5 MHz, in agreement with HYSCORE experiments (Supplementary Information S11b). Even if for the single-quanta transitions there is a severe overlap with the blind spot region, the EDNMR spectra contain a significant set of signals also for frequencies higher than 10 MHz, due to the ^14^N nuclei coupled with different hyperfine coupling constants. This allows us to focus on the spectral regions higher than 10 MHz and to observe informative differences. As for the HYSCORE experiments, Fig. 6c,d do not exhibit remarkable differences; the presence of one covalently attached ligand (L_2_) within the DNA sequence does not show the same coordination sphere of the ligand in the monomeric form. By analyzing the three component mixture (orange line, Fig. 6e), a clear splitting of signal is observed, allowing for differentiation between ^14^N deriving from the native DNA duplex and ^14^N deriving from the monomeric ligand L_2_. The echo-detected field-swept spectra (EDFS) are shown as insets on the top-right of Fig. 6. The HTA pulse was applied to the *B* value indicated on the spectrum (blue arrow). In order to verify the orientation effect of the g-tensor with respect to the EDNMR experiments, a second *B* value was chosen, as shown in Fig. 7. For the g-component centered at 1080 mT the splitting mentioned above is even more pronounced. The signals corresponding to ^14^N nuclei of monomeric ligands (16.2 and 23.7 MHz) are joined by the signal of the ^14^N (duplex, at 30.6 MHz) and by those of the monomeric ligand in the non-covalent mixture (19.2 MHz). In contrast to strongly coupled nuclei, weakly coupled ^1^H nuclei, observed as signals at *ca*. ±51.4 MHz, are not subject to asymmetries depending on the magnetic field positions. This is because the hyperfine coupling of weakly coupled ^1^H nuclei is smaller than the inhomogeneous EPR linewidth. This effect allows us also to differentiate among remote and local hydrogen atoms, as shown for Cu^2+^ in solution at two different observed B-values (Supplementary Information S12). To complete and support this discussion, especially as far as the conformational properties of imidazole ring in the major groove of the DNA duplex with one modified nucleoside are concerned, Molecular Dynamics (MD) simulations have been performed (Supporting Information S14 and S15). Thus, for the label attached onto deoxyadenosine residue at ninth position, as the structure displayed in Fig. 2c, the dihedral angle defined by the four atoms marked in the inset of Figs S14 and S15 was monitored. The distribution of the dihedral angle suggests four conformations with major populations at −180° and +180° and minor populations at −60° and +60°. With respect to the standard *B*-DNA structure, the minor population conformations are buried deeper into the major groove. Only the major conformations at −180° and +180° are more exposed. The flexibility/rigidity of the linker might determine the accessibility and functional efficiency of the imidazole ring in its interaction with Cu^2+^ ions.

## Discussion

Looking at the monomeric structures of the two ligands, the absence of the urea bridge on the L_2_ structure does not affect the binding efficiency. Indeed, the ethyl (alkyl) bridge among the imidazole unit and the nucleoside can still guarantee a strong coordination to the paramagnetic center as that of L_1_, as demonstrated by different EPR parameters. Furthermore, degradation of the urea bridge within more complex oligomers is then avoided by using ligand L_1_, containing exclusively the ethyl bridge attached to the nucleoside. The binding modes identified in this work suggest a preferred affinity to nitrogen nuclei, even if the role of the phosphate backbone remains elusive, with respect to the major/minor grooves of DNA. However, it paves the way for several different approaches, including selective inhibition of binding sites by amplifying or reducing the intercalative efficiency of those ligands (L_1_ or L_2_ in the monomeric form). It seems that the imidazole moiety within the duplex has lower coordination propensity compared to the free monomeric ligand. Furthermore, the EPR/ESR studies strongly suggest a reduced accessibility of the imidazole moiety that can be overcome by using it as intercalating agent.

The overall study proposed in this work suggests that the *non*-covalent mixtures can guarantee the suitable binding affinity towards copper ions. It would be extremely interesting to monitor and follow cleavage and ligation upon different binding modes to identify those nuclei mainly responsible for the catalytic core of DNAzymes/deoxyribozymes, as well as ribozymes and other catalytic nucleic acids. Recently, the impact of tethered imidazole on duplex stabilization has been reported in an aptamer-type construction^32^; such enhanced stabilization could be also key to the *non*-covalent approach illustrated in this work. The modularity in the binding approach has also been reported for terpyridine/aptamer conjugates^33^; a suitable spatial configuration for a substrate within a flexible DNA region is even able to increase the affinity for Cu^2+^ and Fe^3+^ during the oxidation of dopamine to aminochrome. Even for those very recent examples a spectroscopic analysis that can achieve atomistic resolution is severely lacking.

This work paves the way for a series of promising and novel experiments. First of all, the entire set of structures here proposed exhibit natural isotopic abundance; this means that in future, selective coupling can be switched on/off by using ^13^C, ^2^H, ^15^N or ^17^O enriched isotope analogues. For example, the number of transitions for ^14^N/^15^N will greatly simplify the HYSCORE and EDNMR spectra (Supplementary Information S13). Secondly, self-cleaving DNA sequences can be monitored during the cleavage reaction by applying two different approaches: a single modified nucleotide can be mixed to the native DNA sequence and the catalytic effect of Cu^2+^ can be checked in terms of hyperfine couplings with the nuclei in close proximity. For the EPR pulsed experiments, the EDNMR can be further improved by higher-powered ELDOR pulses. The combination frequencies involving the simultaneous excitation of two different nuclei can make the interpretation of spectra more difficult. However, by simulation of the EDNMR spectra, as well as comparison with more conventional ENDOR experiments and integrative combination of DFT data, a description at atomic scale can be obtained. It is possible to state that the loss of affinity for singly labelled oligonucleotides strongly supports a multi-sites binding scenario, as confirmed by signal splittings in the EDNMR spectra. Fascinating opportunities arise for transferring the approach here proposed from the structure to the reactivity of catalytic DNA fragments. Investigations in this direction are ongoing and will be reported in due course.

## Methods

### Synthesis of DNA sequence

Synthesis of L_1_ and L_2_ ligands was performed and adapted from ref.^3^. Solid-phase synthesis of DNA duplexes singly modified with deoxyadenosine-L_1_ (or –L_2_) was carried out according to procedures described elsewhere^32^.

### Sample preparation for EPR experiments

Duplexes have been formed by heating DNA strands at 90 °C for 3 minutes and slowly cooled down to room temperature. For the different pH, three buffers were used: Sodium Acetate (pH 4.00), Cacodylate (pH 7.45), Glycine/NaOH (pH 10.3). 10% (v/v) of glycerol was added before freezing the sample in liquid Nitrogen. With respect to Cu^2+^ solution (CuCl_2_), a molar excess of monomeric ligand or double helix was used, up to an excess of 1:10. 100 mL solutions were used in 4 mm tubes (X-band) and 3 mm tubes (Q-band), respectively.

### EPR experiments

Continuous Wave (CW) X-Band measurements were carried out using an X-band Bruker E500 instrument (9.4 GHz, TE_012_ resonator) equipped with a nitrogen flow cryostat. All CW experiments were recorded at 120 K and with a shot repetition rate of 100 Hz, unless stated otherwise. Pulsed EPR experiments at X-band were performed on a Bruker ELEXYS E-580X-band spectrometer with a SuperX-FT microwave bridge and a Bruker ER EN4118X-MD4 dielectric resonator. Cryogenic temperatures (20 K) were obtained by the use of an Oxford flow cryostat. The field-swept EPR spectra were recorded by electron spin echo (ESE) detection; electron-spin-echo (ESE)-detected EPR experiments were carried out with the pulse sequence: π/2–τ–π–τ–echo. For the X-band experiments the mw pulse lengths t_π/2_ = 16 ns and t_π_ = 32 ns and a τ value of 200 ns were used. A two-step phase-cycle was applied to remove all unwanted echoes.

The Hyperfine Sublevel Correlation (HYSCORE) experiments were carried out using the pulse sequence π/2–τ–π/2–t_1_–π–t_2_–π/2–τ–echo. The time traces of the HYSCORE spectra were baseline corrected using a third-order polynomial, apodized with a Hamming window and zero-filled. After two-dimensional Fourier transformation, the absolute value spectra were calculated. A four-step phase cycle (for X-band experiments) was used to remove unwanted echoes.

The ELDOR-detected NMR (EDNMR) measurements were performed at the Q-band frequency using the pulse sequence HTA–T–π/2–τ–π–τ–echo. A high turning angle (HTA) microwave pulse was applied at the microwave frequency ν_2_. The detection Hahn echo pulse sequence π/2–τ–π–τ–echo was matched with the cavity resonance microwave frequency ν_1_ and the spectra were acquired *via* continuous sweeping of the HTA frequency ν_2_ at fixed B_0_. The length of the HTA pulse was optimized to 9 μs. Between the HTA pulse and the detection sequence a delay τ_d_ of 204 ns was included. In the ESE detection sequence the same pulse lengths and interpulse delay as in the field-swept EPR experiments were applied. To ensure sufficient sensitivity, the echo integration window τ_int_ was set to 500 ns. No phase-cycle was used in the EDNMR experiments. The simulations of the EPR spectra were performed with the Easyspin software package (version 5.2.21)^31^.

### DFT calculations

All DFT calculations for L_1_ and L_2_ ligands bound to Cu^2+^ ions were performed with the ADF (Amsterdam Density Functional) code developed by E. J. Baerends and co-workers^34^. Triple-zeta basis sets have been used throughout. All geometry optimizations were performed *in vacuo* relying on the Generalized Gradient Approximation (GGA) VBP exchange-correlation (XC) potential (VWN + BP: Vosko, Wilk & Nusair^35^ + corrective terms by Becke^36^ for the exchange, and Perdew^37^ for the correlation) with ADF grid precision 6. Subsequently, the computation of hyperfine coupling tensors relied on the use of the B3LYP exchange-correlation potential^38,39^ (20% of Hartree-Fock exchange) turning on the (non-relativistic) ADF option ESR.

### MD simulation

3D structure of Imidazole labelled adenosine monophosphate was generated in Gabedit^40^. Structure was capped at O3′, and phosphate oxygens using methyl groups before geometry optimization. Using the ORCA package^41^, the structure was optimized at restricted Hartree-Fock level of theory using 6–31 g* basis set^42^. Using ambertools18, AM1-BCC charges were calculated and generalized amber force field topology compatible with gromacs2018.2^43^ package was generated for labelled deoxyadenosine. Structure for duplex DNA was generated using make-na server (http://structure.usc.edu/make-na/server.html). Imidazole-derivative molecule was fitted onto deoxyadenosine residue, using the purine ring atoms (only carbon and nitrogen) for the least square fitting, and coordinates of imidazole ring were transferred to pdb file creating the labelled DNA molecule. DNA molecule was modelled using parmbsc1 forcefield^44^. Labeled duplex was placed in a dodecahedron box with 1.0 nm distance between the box walls and DIMA. Simulation box was solvated with tip3p water molecules and salt concentration was set to 0.15 M using Na^+^ and Cl^−^ ions. Simulation system was energy minimized using steepest descent algorithm until the largest force was smaller than 1000 kJ/mol/nm, followed by temperature equilibration to 300 K in 100 ps using Berendsen thermostat with a tau-t of 0.1 ps. Pressure was equilibrated to 1 atm in 1 ns using Berendsen barostat and temperature was regulated using velocity-rescaling thermostat at 300 K. Using the equilibrated structure, three 100 ns production run simulations were started in which temperature was regulated using velocity-rescaling thermostat and pressure with Parrinello-Rahman barostat at 300 K and 1 atm using tau-t of 1 ps and tau-p of 2 ps. Structures were saved every 10 ps. Further details of MD analysis are provided in Supporting Information S16.

## Supplementary information

Supplementary Information

## References

[1] Hemschemeier A, Happe T (2018). The plasticity of redox cofactors: from metalloenzymes to redox-active DNA. Nature Reviews.

[2] Pugh GC, Bruns JR, Howorka S (2018). Comparing proteins and nucleic acids for next-generation biomolecular engineering. Nature Reviews.

[3] Curtis EA, Bartel DP (2005). New catalytic structures from an existing ribozyme. Nature Structural and Molecular Biology.

[4] Lodyga-Chruscinska E (2010). Histamine modified 2′-deoxyriboadenosine – Potential copper binding site in DNAzyme. J. Inorg. Biochem..

[5] Borowska J, Sierant M, Sochacka E, Sanna D, Lodyga-Chruscinska E (2015). DNA binding and cleavage studies of copper(II) complexes with 2′-deoxyadenosine modified histidine moiety. J. Biol. Inorg. Chem..

[6] Lodyga-Chruscinska E, Sierant M, Pawlak J, Sochacka E (2013). Physicochemical and biological properties of nucleosides modified with an imidazole ring and their copper complexes. QScience Connect.

[7] Yang Z (2012). ESR spectroscopy identifies inhibitory Cu2+ sites in a DNA-modifying enzyme to reveal determinants of catalytic specificity. PNAS.

[8] Wang Y, Liu E, Lam CH, Perrin DM (2018). A densely modified M2+-independent DNAzyme that cleaves RNA efficiently with multiple catalytic turnover. Chem. Sci..

[9] Engelhard DM, Meyer A, Berdhaeuser A, Schiemann O, Clever GH (2018). Di-copper(II) DNA G-quadruplex as EPR distance rulers. Chem. Comm..

[10] Chikira M, Ng CH, Palaniandavar M (2015). Interaction of DNA with simple and mixed ligand copper(II) complexes of 1,10-phenanthrolines as studied by DNA-fiber EPR spectroscopy. Int. J. Mol. Sci..

[11] Schwizer F (2018). Artificial metalloenzymes: reaction scope and optimization strategies. Chem. Rev..

[12] Liu M, Chang D, Li Y (2017). Discovery and biosensing applications of diverse RNA-cleaving DNAzymes. Acc. Chem. Res..

[13] Luke Ward L, Plakos K, De Rose VJ (2014). Nucleic Acid Catalysis: Metals, Nucleobases, and Other Cofactors. Chem. Rev..

[14] Lada ZG (2017). Probing the electronic structure of a copper(II) complex by CW- and pulse-EPR spectroscopy. Dalton Trans..

[15] Dancs A, Selmeczi K, May NV, Gaja T (2018). On the copper(II) binding of asymmetrically functionalize tripodal peptides: solution equilibrium, structure and enzyme mimicking. New J. Chem..

[16] Mondal Satyajit, Chakraborty Moumita, Mondal Antu, Pakhira Bholanath, Blake Alexander J., Sinn Ekkehard, Chattopadhyay Shyamal Kumar (2018). Cu(ii) complexes of a tridentate N,N,O-donor Schiff base of pyridoxal: synthesis, X-ray structures, DNA-binding properties and catecholase activity. New Journal of Chemistry.

[17] Sicoli G (2016). Fine-tuning of a radical-based reaction by radical *S*-Adenosyl Methionine (SAM) tryptophan lyase. Science.

[18] Molle T (2016). Unanticipated coordination of tris buffer to the Radical SAM cluster of the RimO methylthiotransferase. J. Biol. Inorg. Chem..

[19] Le Breton N (2017). Using hyperfine electron paramagnetic resonance spectroscopy to define the proton-coupled electron transfer reaction at Fe-S cluster N2 in respiratory complex I. J. Am. Chem. Soc..

[20] Cox N, Nalepa A, Lubitz W, Savitsky A (2017). ELDOR-detected NMR: A general and robust method for electron-nuclear heprfine spectroscopy?. J. Magn. Reson..

[21] Hetske T, Bowen AM, Prisner TF (2017). ELDOR-detected NMR at Q-band. Appl. Magn. Reson..

[22] Schosseler P, Wacker T, Schweiger A (1994). Pulsed ELDOR detected NMR. Chem. Phys. Lett..

[23] Ritterskamp N (2017). Understanding the coordination modes of [Cu(acac)2(imidazole)n = 1,2] adducts by EPR, ENDOR, HYSCORE an DFT analysis. Inorg. Chem..

[24] Wili C, Jeschke G (2018). Chirp echo Fourier transform EPR-detected NMR. J. Magn. Reson..

[25] Slota M (2018). Magnetic edge states and coherent manipulation of graphene nanoribbons. Nature.

[26] Rapatskiy L (2015). Characterization of oxygen bridged manganese model complexes using multifrequency ^17^O-hyperfine EPR spectroscopies and density functional theory. J. Phys. Chem. B.

[27] Zhan G, Zhong W, Wei Z, Liu Z, Liu X (2017). Roles of phenol groups and auxiliary ligand of copper (II) complexes with tetradentate ligands in the aerobic oxidation of benzyl alcohol. Dalton Trans..

[28] Silverman SK (2015). Pursuing DNA catalysts for protein modification. Acc. Chem. Res..

[29] Silverman SK (2009). Deoxyribozymes: selection design and serendipity in the development of DNA catalysts. Acc. Chem. Res..

[30] Zhang W, Liu M, Lee C, Salena BJ, Li Y (2018). Serendipitous discovery of a guanine-rich DNA molecule with a highly stable structure in urea. Scientific Reports.

[31] http://www.easyspin.org/ [Easyspin software package (version 5.2.21)].

[32] Verdonck, L. *et al*. Tethered imidazole mediated duplex stabilization and its potential for aptamer stabilization *Nucleic Acids Res*., 10.1093/nar:gky1062 (2018). 10.1093/nar/gky1062 PMC6294506 30418582

[33] Biniuri Y (2018). Cu2+ or Fe3+ terpyridne/aptamer cnjugates: nucleoapzymes catalyzing the oxidation of dopamine to aminochrome *ACS*. Catalysis.

[34] Velde GT, Baerends EJ (1992). Numerical integration of polyatomic systems. J. Comput. Phys..

[35] Vosko SH, Wilk L, Nusair M (1980). Accurate spin-dependent electron liquid correlation energies for local spin density calculations: a critical analysis. Canadian Journal of Physics.

[36] Becke AD (1988). Density-functional exchange-energy approximation with correct asymptotic behavior. Phys. Rev. A.

[37] Perdew JP (1986). Density-functional approximation for the correlation energy of the inhomogeneous electron gas. Phys. Rev. B.

[38] Becke AD (1993). A new mixing of Hartree-Fock and local density functional theories. J. Chem. Phys..

[39] Lee CT, Yang WT, Parr RG (1998). Development of the Colle-Salvetti correlation-energy formula into a functional of the electron density. Phys. Rev. B..

[40] Allouche Abdul-Rahman (2010). Gabedit-A graphical user interface for computational chemistry softwares. Journal of Computational Chemistry.

[41] Neese F (2012). The ORCA program system, Wiley Interdiscip. Rev.: Comput. Mol. Sci..

[42] Bykov D (2015). Efficient implementation of the analytic second derivatives of Hartree-Fock and hybrid DFT energies: a detailed analysis of different approximations. Mol. Phys..

[43] Hehre WJ, Ditchfield R, Pople JA (1972). Self—Consistent Molecular Orbital Methods. XII. Further Extensions of Gaussian—Type Basis Sets for Use in Molecular Orbital Studies of Organic Molecules. J. Chem. Phys..

[44] Ivani I (2016). Pambsc1: a refined force field for DNA simulations. Nature Methods.

