# Supplementary material for: A trade-off for covalent and intercalation binding modes: a case study for Copper (II) ions and singly modified DNA nucleoside
Source: Sci Rep. 2019 Aug 29;9:12602. doi: 10.1038/s41598-019-48935-2 (PMC6715747; doi:10.1038/s41598-019-48935-2)
Supplement: Supplementary file 1 — Supplementary Information [file 41598_2019_48935_MOESM1_ESM.pdf]

Supplementary information

A trade-off for covalent and intercalation binding modes: a case study for Copper (II) ions and singly modified DNA nucleoside

Jean-Marie Mouesca,[1] Hania Ahouari,[2] Sarath Chandra Dantu,[3] Giuseppe Sicoli[2]*

[1]Grenoble University, CEA, CNRS, INAC−SyMMES, F−38000 Grenoble, France. [2]CNRS UMR 8516, Lille University, LASIR Institute−Bâtiment C5, Avenue Paul Langevin, F−59655 Villeneuve d'Ascq, France. [3]Department of Computer Science – Synthetic Biology Theme, Brunel University London, Kingston Lane, Uxbridge UB8 3PH, London, United Kingdom.

*Corresponding author: giuseppe.sicoli@univ-lille.fr

S1. CW EPR/ESR spectra for mixture ligand L_1 (ethyl-urea bridge):Cu^{2+} (2:1) at four pH values.
S2. CW EPR/ESR spectra for different ratio ligand/copper for ligand L_2 (ethyl bridge) at pH 7.45.
S3. CW EPR/ESR spectra for different ratios (ligand/Cu2+) for L_1 (ethyl-urea bridge) at pH 7.45
S4. Tables of computed hyperfine couplings (DFT) and EasySpin simulation. In these tables six complexes Cu2+/ligand are summed up: for two different ligands, three different solutions have been used at pH 4.00, 7.45 and 10.3, respectively.
S5. Structures derived by DFT computational studies.
S6. CW EPR/ESR spectra for monomeric L_2/Cu^{2+} (5:1) at pH 7.45 in H_2O and D_2O.
S7. HYSCORE spectra for monomeric form of Cu2+/monomeric L1 (ratio 1:2) at pH 4.00.
S8. HYSCORE spectra for monomeric form of Cu2+/monomeric L1 (ratio 1:2) at pH 7.45.
S9. HYSCORE spectra for monomeric form of Cu2+/monomeric L1 (ratio 1:2) at pH 10.3.
S10. Echo-FS spectra recorded at 34 GHz for the following samples: *a*) $CuCl_2{\cdot}2H_2O$ in H2O; *b*) Monomeric L_2 ligand ('ethyl-bridge') with $CuCl_2{\cdot}2H_2O$ in a cacodylated buffer solution (pH 7.45); *c*) Native (unmodified) DNA sequence with $CuCl_2{\cdot}2H_2O$ in a cacodylated buffer solution (pH 7.45), using an excess 5:1; *c*) single modified DNA sequence (L_2 ligand covalently attached) with $CuCl_2{\cdot}2H_2O$ in a cacodylated buffer solution (pH 7.45), using an excess 5:1; Monomeric L_2 ligand mixed with native DNA sequence (NON-covalently attached) with $CuCl_2{\cdot}2H_2O$ in a cacodylated buffer solution (pH 7.45), using an excess 5:1.
S11. EDNMR spectra for monomeric form of L2/copper(II) complexes at pH 7.45.
S12. EDNMR spectra for $CuCl_2{\cdot}2H_2O$ in a cacodylated buffer solution (pH 7.45); orientation selection experiments and zooming on the *blind spot* regions.
S13. Number of relative transitions for 14N and for 15N. The single-quantum transitions for the ^{15}N simplified tremendously the EPR spectrum and especially the HYSCORE spectrum.
S14. Position and rotation of the imidazole labelled residue deoxyadenosine in the major groove is shown with respect to the standard *B*-DNA in surface representation; for the four main populations the imidazole moiety is partially or completely exposed to the major groove.
S15. Rotation of imidazole ring in the molecular dynamics (MD) simulations. Dihedral angle was calculated along the plane of atoms marked with red circles (*top-left*) and its distribution is shown in grey.
S16. Detailed Molecular Dynamics (MD) analysis.

Supplementary information S1. CW EPR/ESR spectra for mixture ligand L_1 (ethyl-bridge):Cu^{2+} (2:1) at four pH values

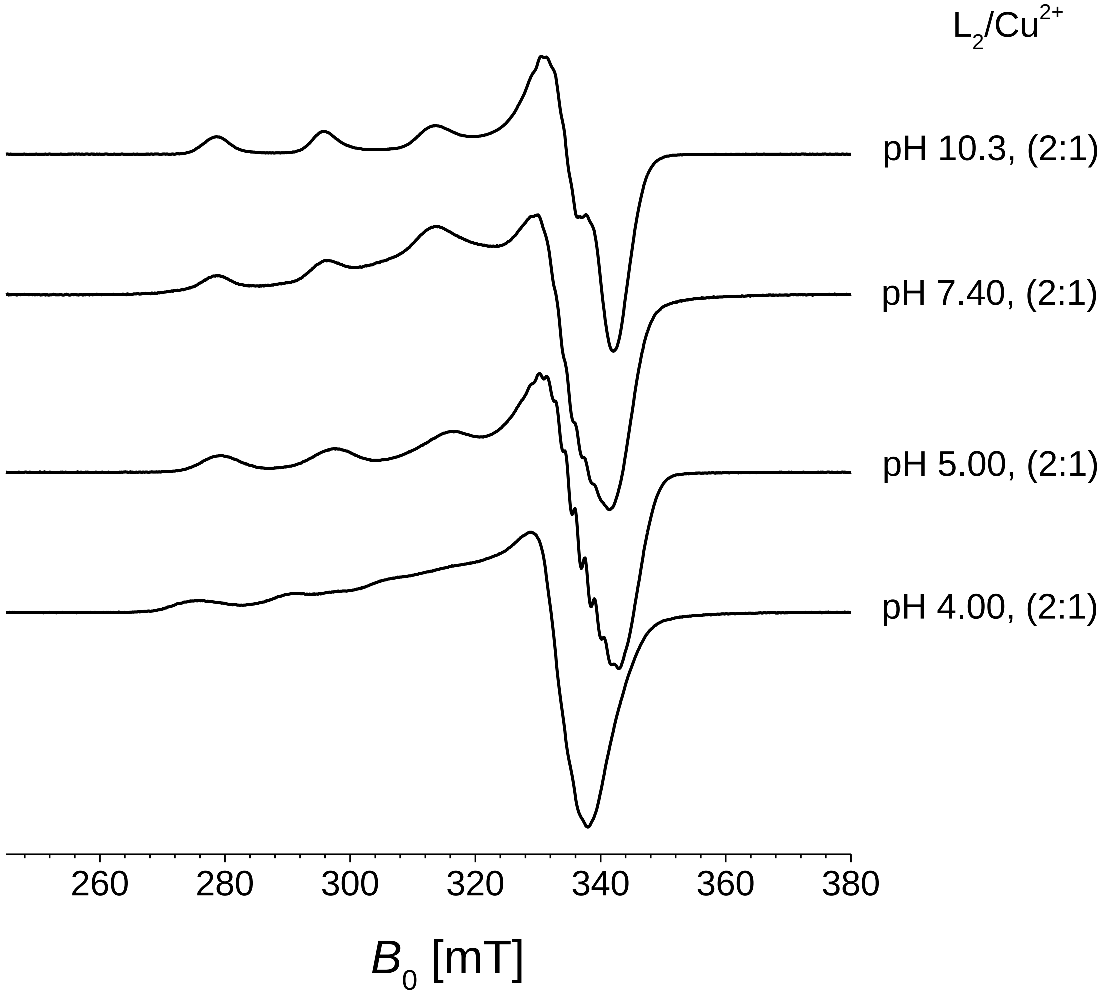

Supplementary information S2. CW EPR/ESR spectra for different ratios (ligand/Cu2+) for L_2 (ethyl-bridge) at pH 7.45

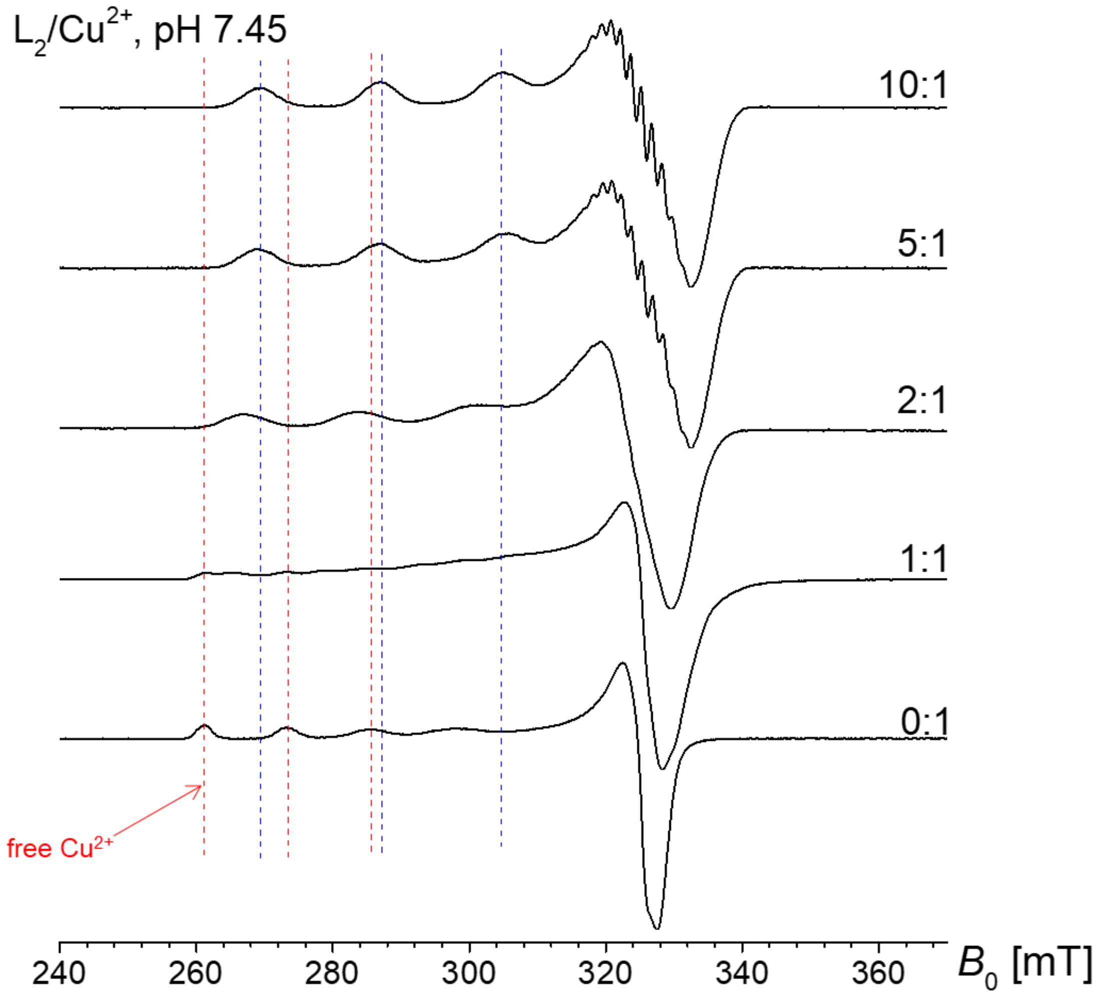

Supplementary information S3. CW EPR/ESR spectra for different ratios (ligand/Cu2+) for L_1 (ethyl-urea bridge) at pH 7.45

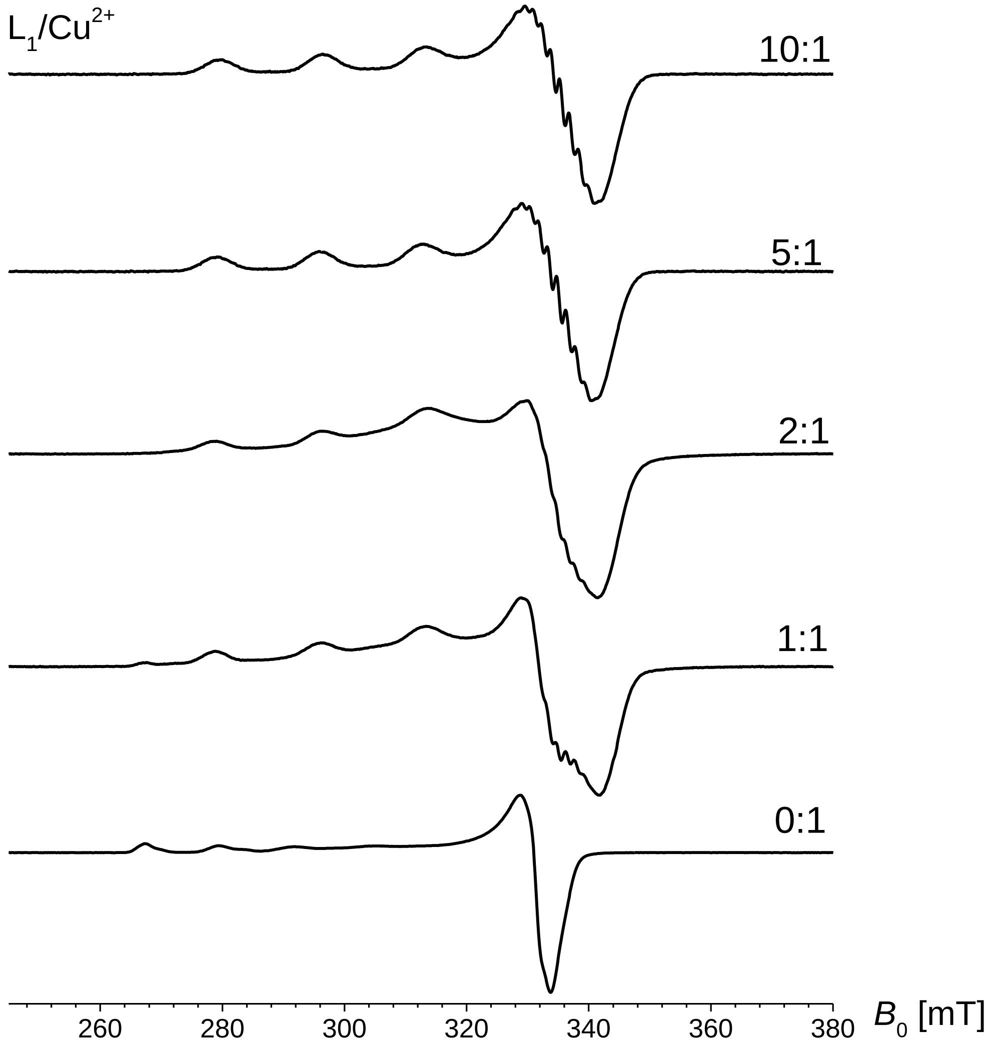

Supplementary information S4. DFT data: summary of hyperfine couplings and comparison with parameters optimized for spectra simulation (EasySpin software)

HisdA (L2), pH 4.00

**Nucleus**	**A (DFT, *MHz*)**	**A (EasySpin, *MHz*)**
^{63}Cu	–253.3<br>231.9<br>511.3<br>a_{iso} 162.9	–106.7<br>106.9<br>517.1<br>a_{iso} 172.4
^{1}H	–7.6<br>–5.6<br>0.9<br>a_{iso} –4.0	–30.9<br>0.1<br>21.9<br>a_{iso} –2.9
^{1}H	8.1<br>9.5<br>20.6<br>a_{iso} 12.8	7.7<br>11.3<br>6.5<br>a_{iso} 8.5
^{14}N	16.6<br>16.8<br>26.8<br>a_{iso} 20.1	17.3<br>–35.7<br>59.3<br>a_{iso} 13.7
^{14}N	0.7<br>1.1<br>7.8<br>a_{iso} 3.2	2.0<br>7.4<br>1.8<br>a_{iso} 3.8
^{14}N	16.5<br>17.0<br>24.3<br>a_{iso} 19.3	43.6<br>–48.2<br>65.8<br>a_{iso} 20.4

HisdA (L2), pH 7.45

**Nucleus**	**A (DFT, *MHz*)**	**A (EasySpin, *MHz*)**
^{63}Cu	-253.3<br>231.9<br>511.28<br>a_{iso} 162.9	–63.2<br>61.4<br>527.5<br>a_{iso} 175.2
^{1}H	–7.3<br>–5.6<br>0.9<br>a_{iso} -4.0	–50.6<br>11.2<br>16.3<br>a_{iso} –7.7
^{1}H	8.1<br>9.5<br>20.6<br>a_{iso} 12.8	2.2<br>–0.3<br>22.3<br>a_{iso} 8.1
^{14}N	16.6<br>16.8<br>26.8<br>a_{iso} 20.1	18.5<br>38.2<br>20.4<br>a_{iso} 25.7
^{14}N	0.7<br>1.1<br>7.8<br>a_{iso} 3.2	–2.4<br>–1.3<br>11.7<br>a_{iso} 2.7
^{14}N	16.5<br>17.0<br>24.3<br>a_{iso} 19.3	43.2<br>–47.9<br>64.4<br>a_{iso} 19.9

HisdA (L2), pH 4.00	
**g**	**g_{strain}**
2.0345(8)	0.0058(8)
2.0751(6)	0.0072(2)
2.2542(2)	0.0343(9)

HisdA (L2), pH 7.45	
**g**	**g_{strain}**
2.0277(4)	0.0077(2)
2.0654(3)	0.0078(1)
2.2565(8)	0.0578(4)

**HisdA (L2), pH 10.3**		
**Nucleus**	**A (DFT, *MHz*)**	**A (EasySpin, *MHz*)**
^{63}Cu	–611.6<br>3.5<br>47.2<br>a_{iso} –187.3	–8.9<br>78.5<br>–563.1<br>a_{iso} –164.5
^{14}N	30.0<br>30.9<br>41.5<br>a_{iso} 34.2	–26.9<br>75.4<br>32.2<br>a_{iso} 26.9
^{14}N	21.4<br>22.0<br>30.3<br>a_{iso} 24.54	51.5<br>76.6<br>37.3<br>a_{iso} 55.1
^{14}N	13.4<br>13.8<br>20.6<br>a_{iso} 15.9	14.5<br>25.8<br>34.8<br>a_{iso} 25.1
^{14}N	19.2<br>19.6<br>29.8<br>a_{iso} 22.9	12.0<br>32.9<br>42.9<br>a_{iso} 29.3

**HisdA (L2), pH 10.3**	
**g**	$\mathbf{g_{strain}}$
2.0546(1)	0.0072(0)
2.0564(1)	0.0087(0)
2.2543(9)	0.0346(3)

**HisdA (L1), pH 4.00**		
**Nucleus**	A (DFT, *MHz*)	A (EasySpin, *MHz*)
^{63}Cu	–729.6<br>–51.5<br>3.5<br>a_{iso} –259.2	–46.5<br>39.2<br>–567.3<br>a_{iso} –191.5
^{1}H	2.0<br>2.5<br>4.5<br>a_{iso} 3.0	–3.1<br>–1.9<br>8.9<br>a_{iso} 3.4
^{1}H	–10.5<br>–5.4<br>5.9<br>a_{iso} –3.3	–13.7<br>–5.5<br>3.2<br>a_{iso} –5.3
^{14}N	42.4<br>43.5<br>57.8<br>a_{iso} 47.9	34.1<br>71.1<br>56.72<br>a_{iso} 52.0
^{14}N	2.5<br>2.8<br>3.6<br>a_{iso} 2.9	5.6<br>–4.8<br>5.4<br>a_{iso} 2.1
^{14}N	40.1<br>41.4<br>58.0<br>a_{iso} 46.5	60.3<br>88.9<br>50.3<br>a_{iso} 66.5

**HisdA (L1), pH 7.45**		
**Nucleus**	A (DFT, *MHz*)	A (EasySpin, *MHz*)
^{63}Cu	-658.1<br>-66.6<br>53.5<br>a_{iso} –223.7	–42.7<br>46.5<br>–542.29<br>a_{iso} –181.4
^{1}H	2.04<br>2.46<br>4.47<br>a_{iso} 2.99	–1.8<br>10.1<br>2.2<br>a_{iso} 3.5
^{1}H	–10.5<br>–5.4<br>5.9<br>a_{iso} –3.3	–24.7<br>–6.6<br>11.5<br>a_{iso} –2.2
^{14}N	27.1<br>27.8<br>37.8<br>a_{iso} 30.88	68.8<br>57.7<br>–0.1<br>a_{iso} 42.1
^{14}N	1.7<br>2.0<br>2.5<br>a_{iso} 2.1	18.3<br>–10.2<br>1.9<br>a_{iso} 2.1
^{14}N	28.9<br>29.5<br>37.9<br>a_{iso} 32.1	39.4<br>42.5<br>35.7<br>a_{iso} 39.2

**HisdA (L1), pH 4.00**	
**g**	**g_{strain}**
2.0558(1)	0.0054(2)
2.0530(2)	0.00124(2)
2.2491(1)	0.0454(2)

**HisdA (L1), pH 7.45**	
**g**	**g_{strain}**
2.0366(3)	0.0097(3)
2.0778(3)	0.0090(3)
2.2604(1)	0.0422(8)

**HisdA (L1), pH 10.3**		
**Nucleus**	**A (DFT, *MHz*)**	**A (EasySpin, *MHz*)**
^{63}Cu	-647.4<br>-2.4<br>97.7<br>a_{iso} –184.0	24.3<br>47.2<br>–568.2<br>a_{iso} –165.6
^{14}N	45.8<br>46.7<br>56.7<br>a_{iso} 49.7	37.4<br>47.4<br>44.4<br>a_{iso} 43.0
^{14}N	45.7<br>46.6<br>56.6<br>a_{iso} 49.6	34.4<br>71.4<br>40.2<br>a_{iso} 48.7
^{14}N	35.8<br>36.6<br>49.4<br>a_{iso} 40.6	55.3<br>35.9<br>41.9<br>a_{iso} 44.4
^{14}N	35.8<br>36.6<br>49.4<br>a_{iso} 40.6	48.7<br>30.0<br>47.9<br>a_{iso} 42.2

**HisdA (L1), pH 10.3**	
**g**	**g_{strain}**
2.0501(6)	0.0029(7)
2.0706(3)	0.0079(7)
2.2596(0)	0.0293(8)

**Cu^{2+} (free)**	
**g**	**g_{strain}**
2.0916(2)	0.0094(4)
2.0739(1)	0.0079(2)
2.4137(3)	0.0199(7)

Supplementary information S5. Structures derived by DFT computations.

L_2/Cu^{2+}, pH 4.00

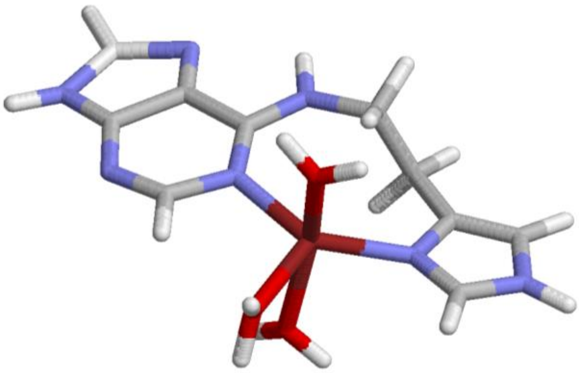

L_2/Cu^{2+}, pH 7.45

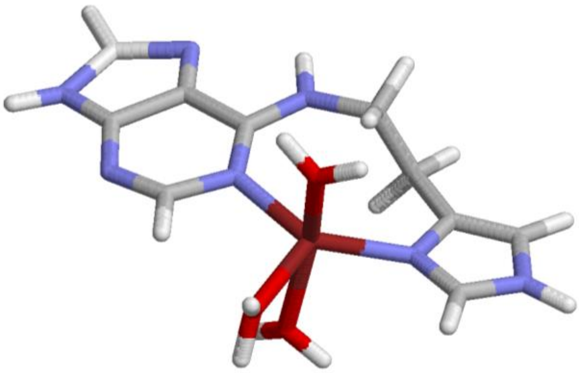

L_2/Cu^{2+}, pH 10.3

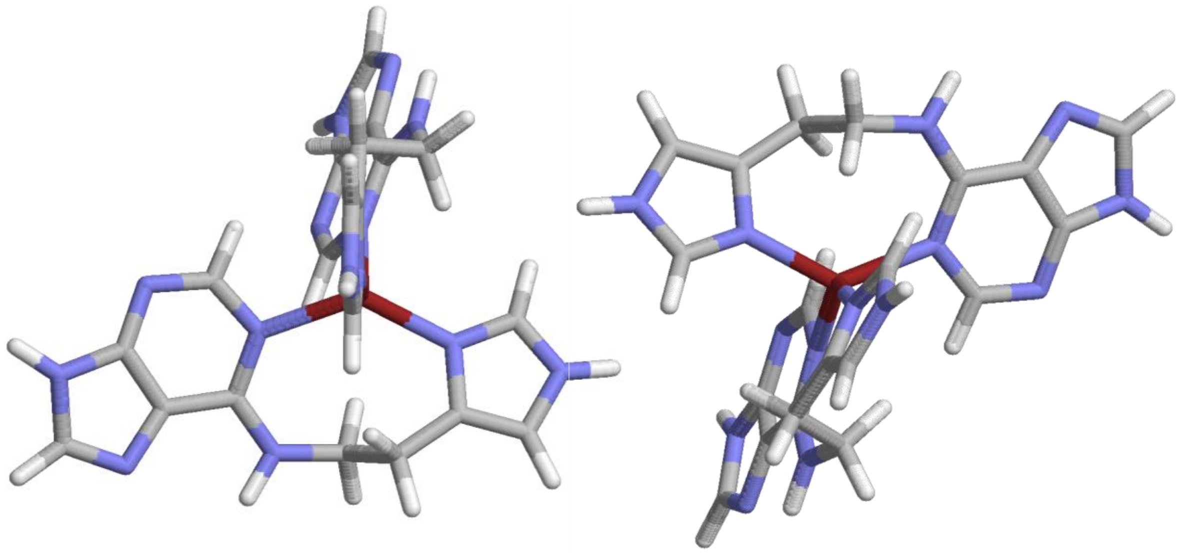

L$_1$/Cu^{2+}, pH 4.00

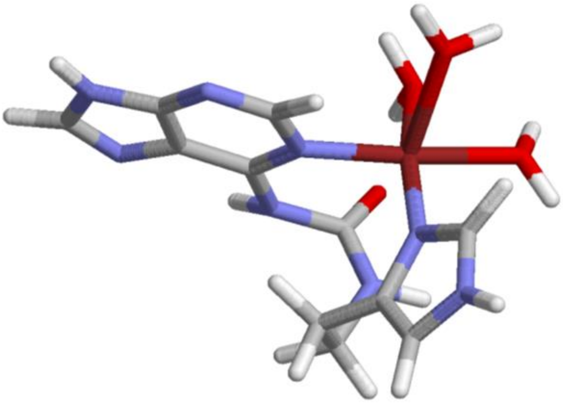

L$_1$/Cu^{2+}, pH 7.45

a)

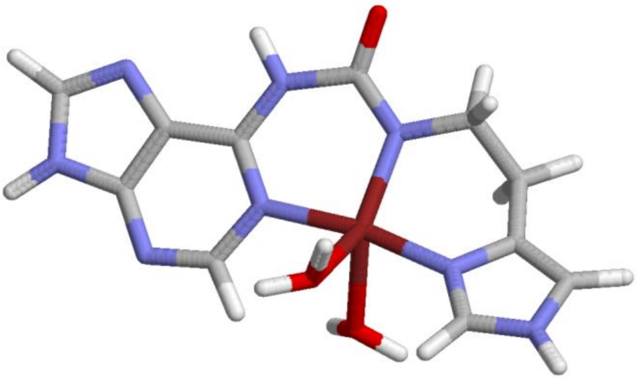

b)

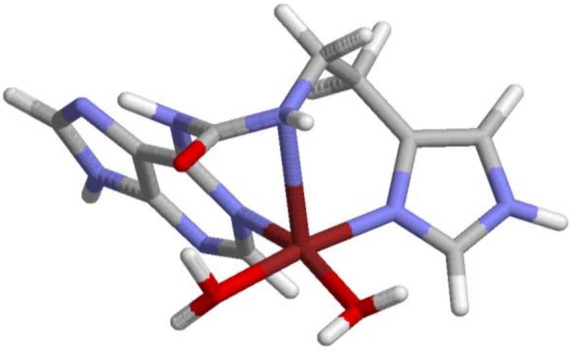

L_1/Cu^{2+}, pH 10.3

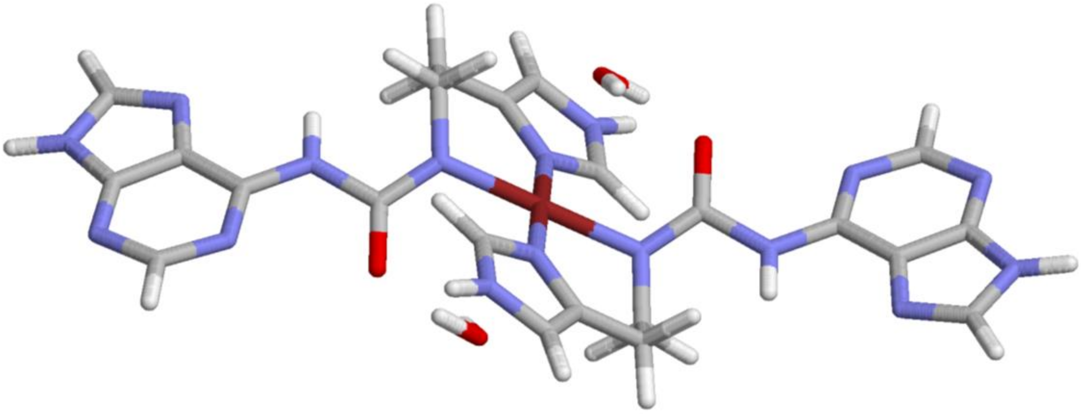

Supplementary information S6. CW EPR/ESR spectra for monomeric L_2/Cu^{2+} (5:1) at pH 7.45 in H_2O and D_2O.

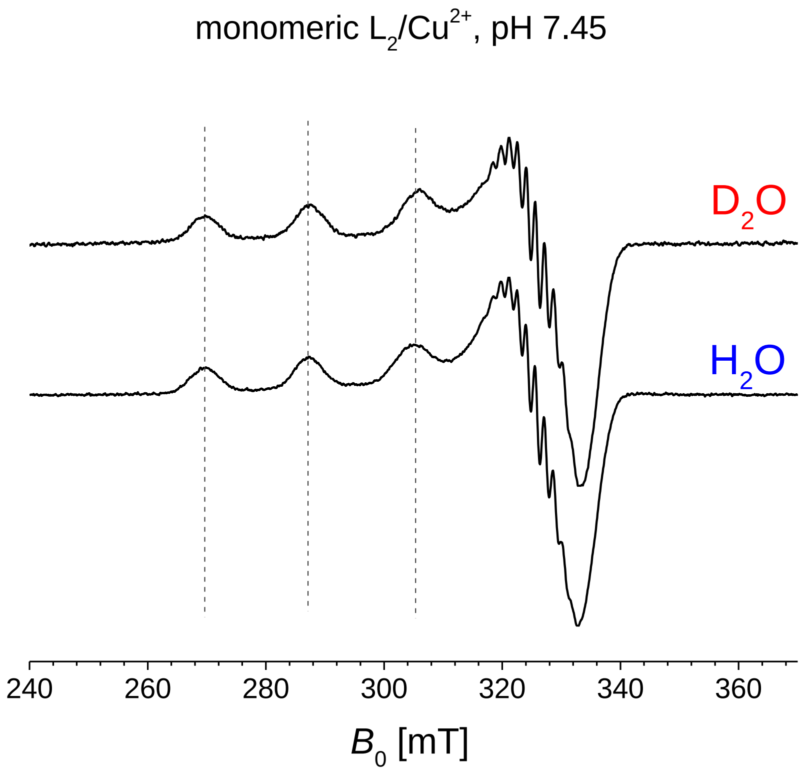

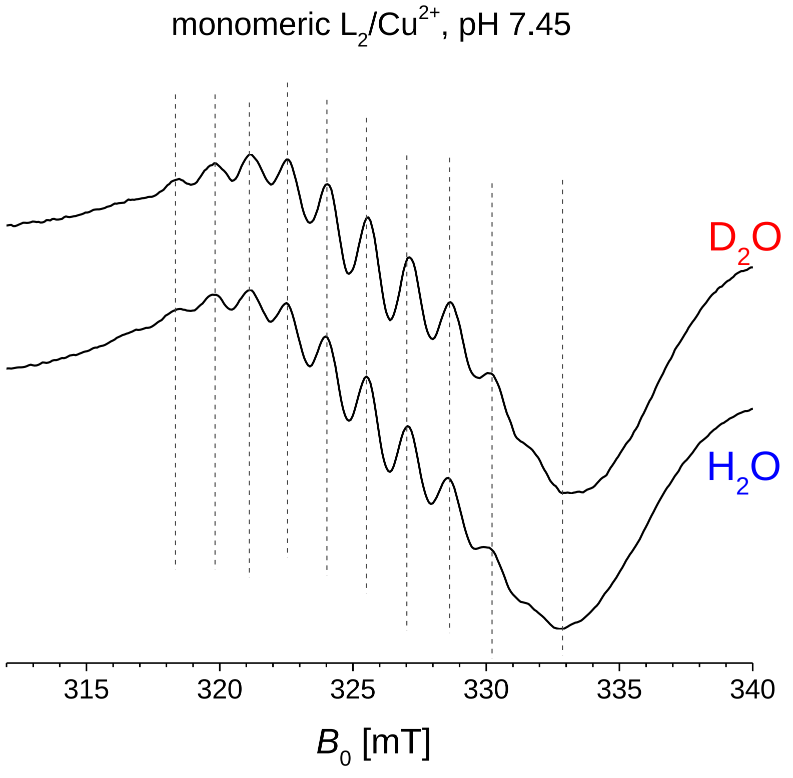

Supplementary information S7

CuL1_pH_4.00_ratio_1:2

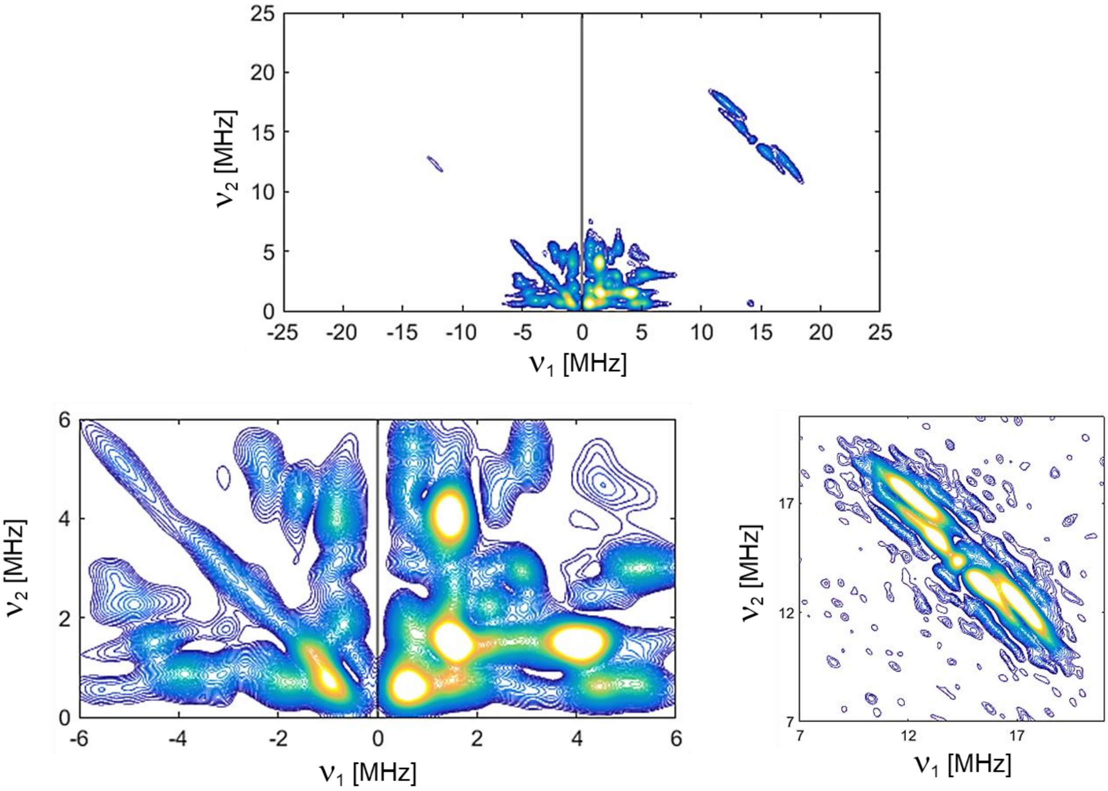

Supplementary information S8

CuL1_pH_7.45_ratio_1:2

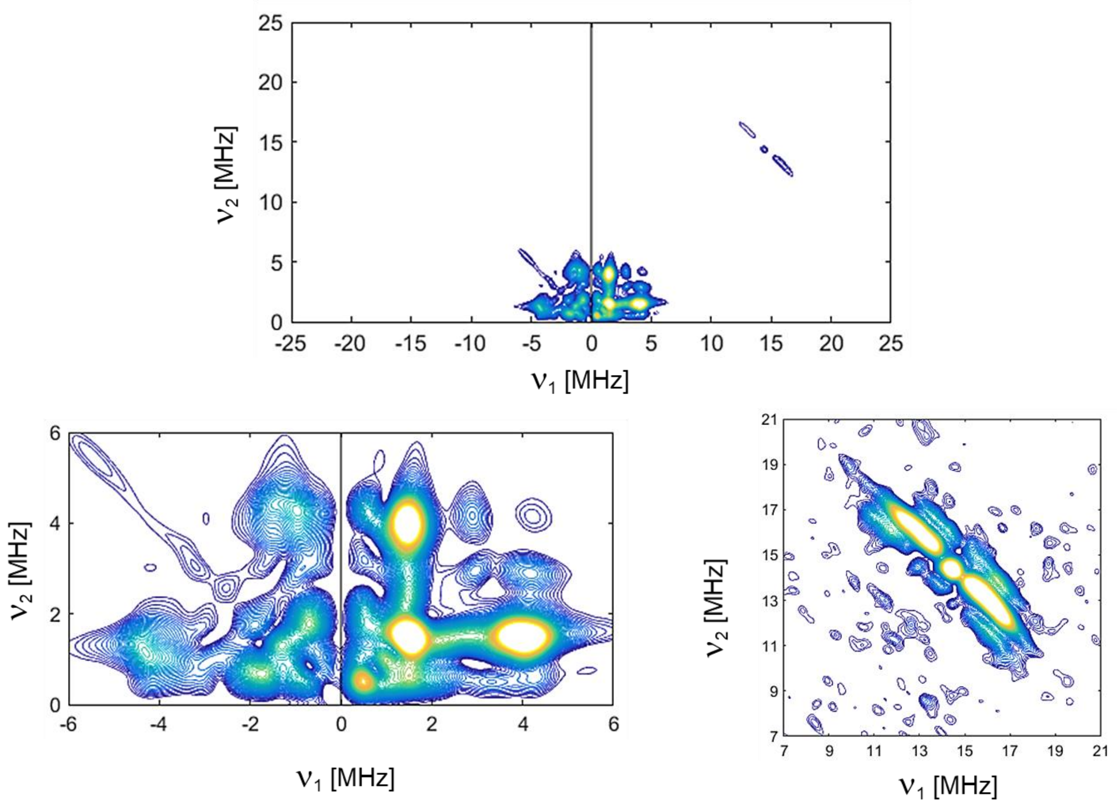

Supplementary information S9.

CuL1_pH_10.3_ratio_1:2

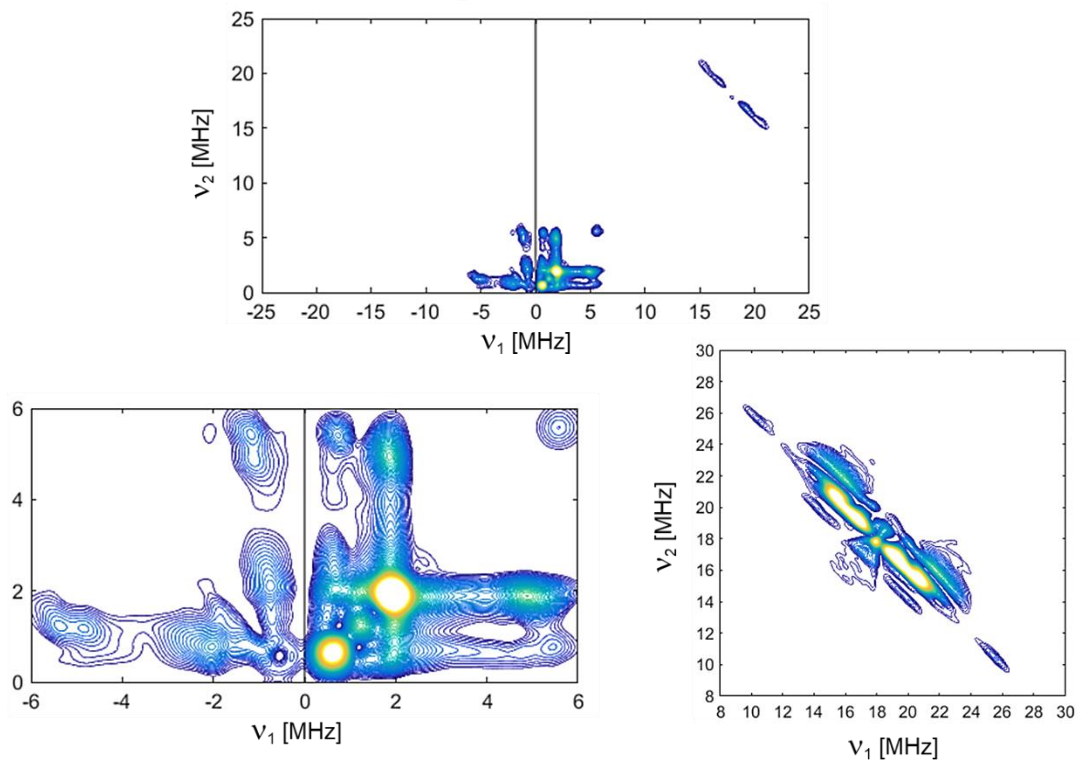

Supplementary information S10.

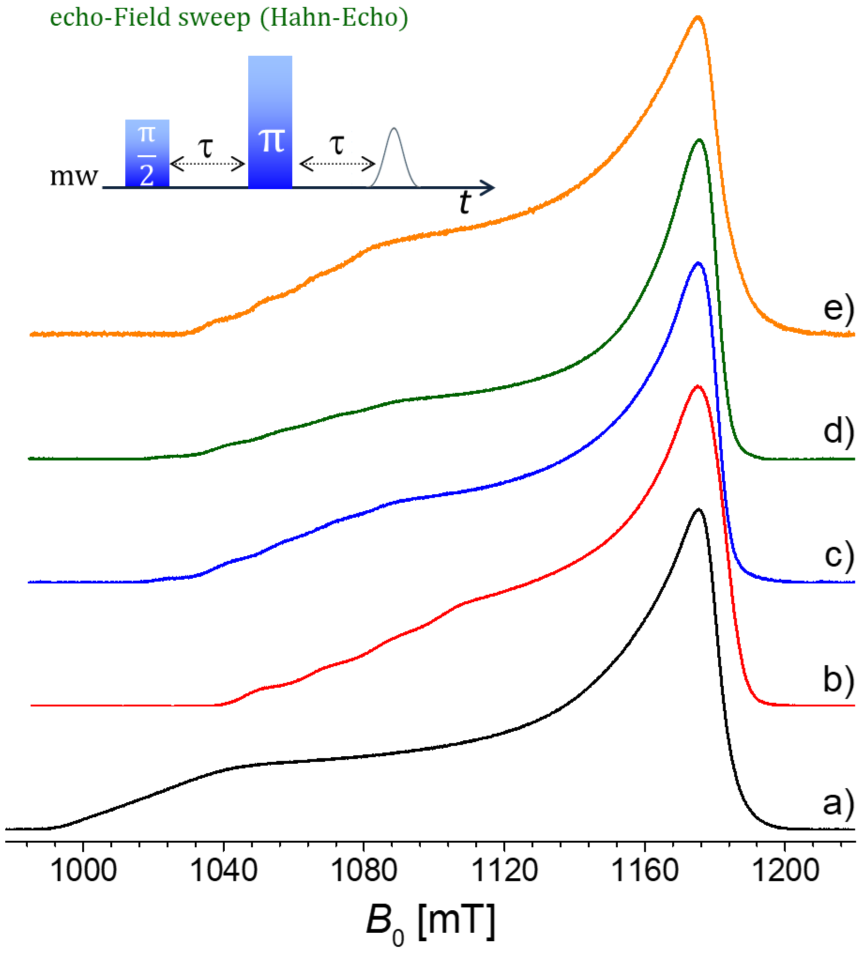

Supplementary information S11.

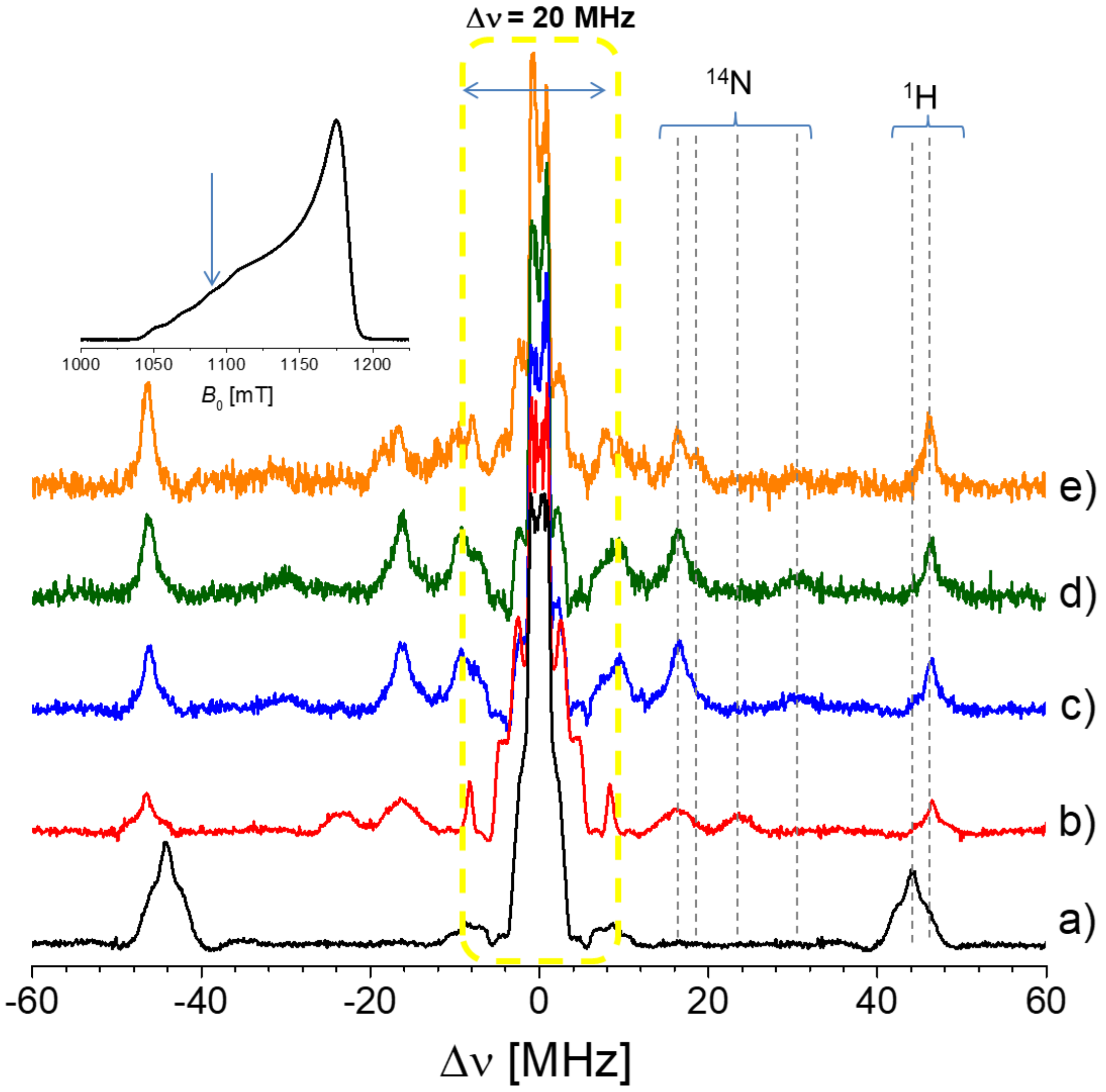

Supplementary information S12.

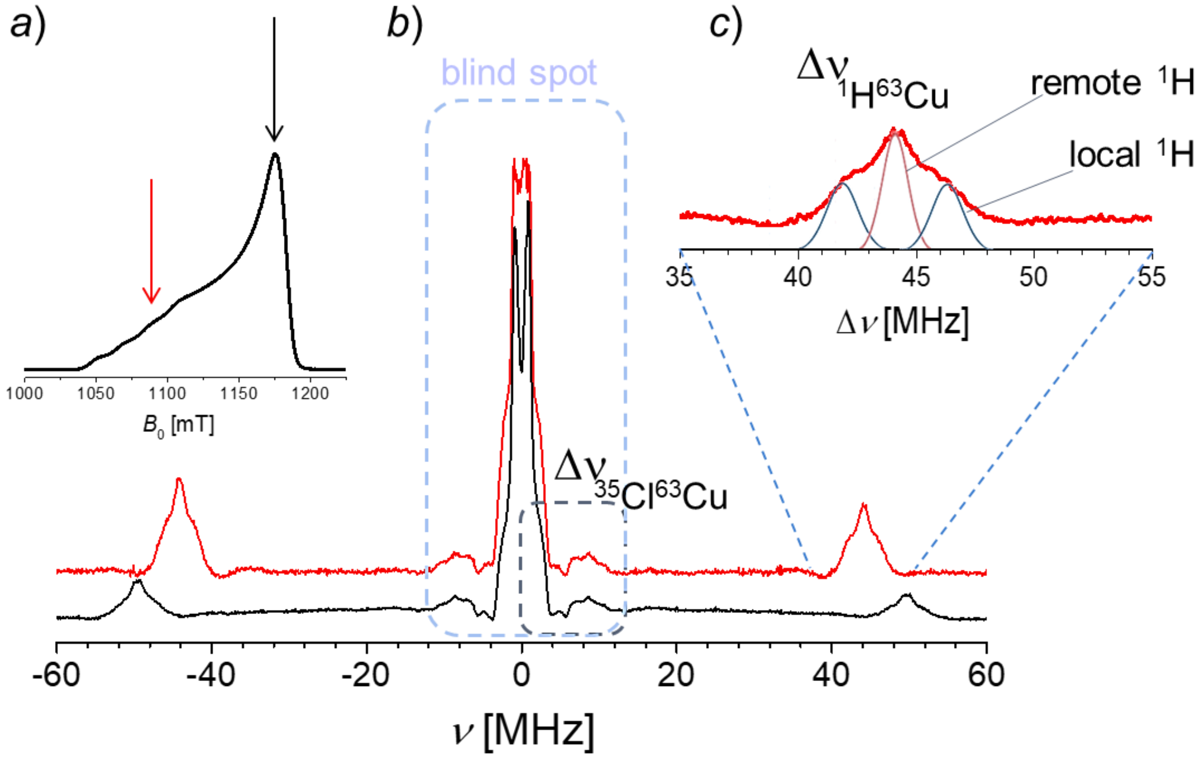

Supplementary information S13.

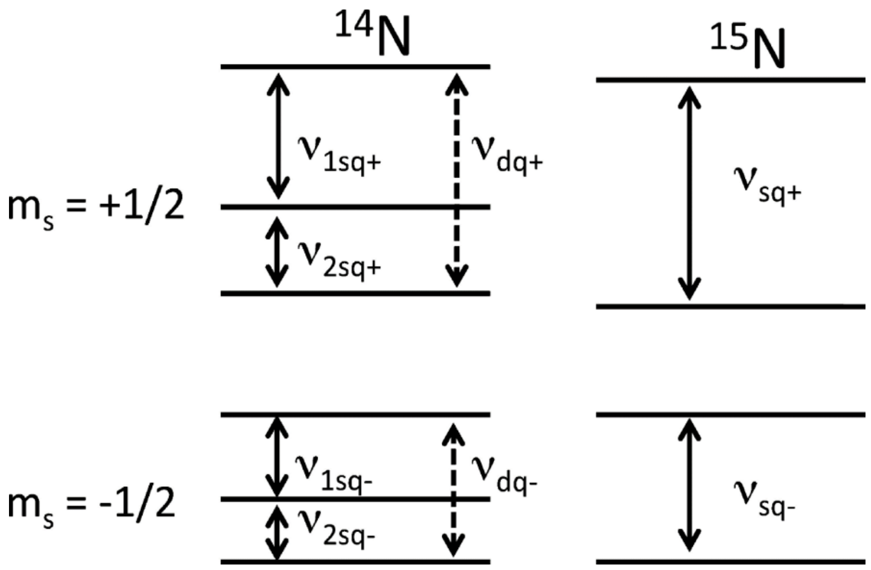

Supplementary information S14.

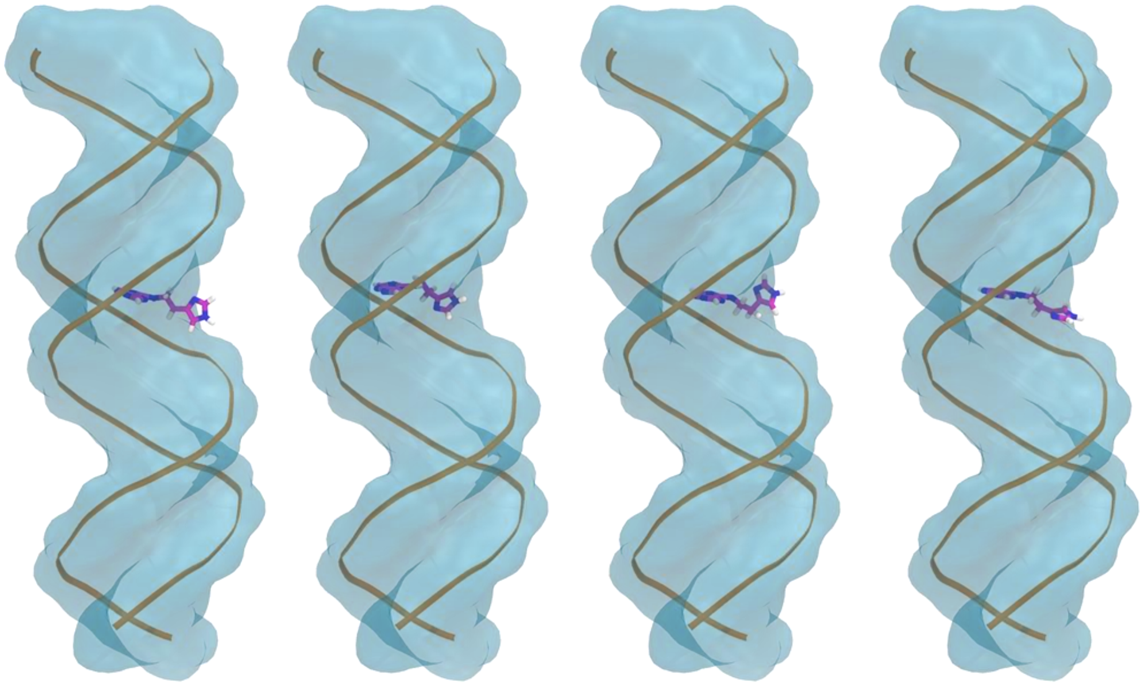

-180°　　-60°　　60°　　180°

Supplementary information S15. MD analysis of the duplex singly modified with the L2 ligand.

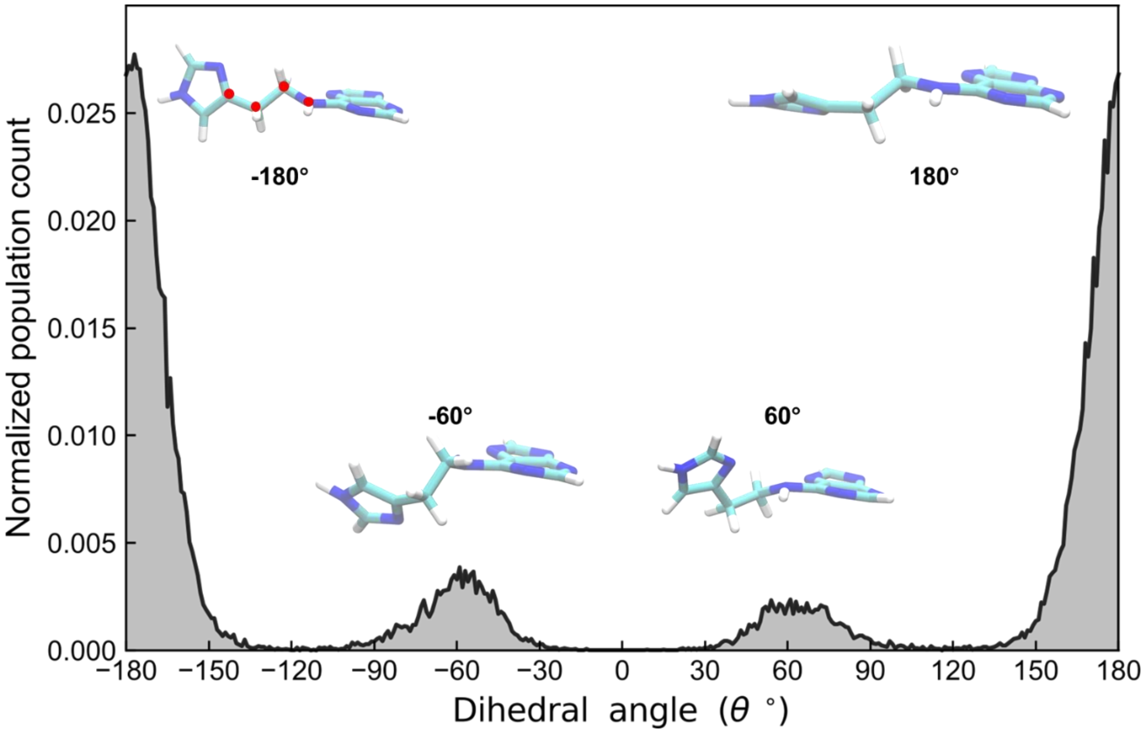

Supplementary information S16. Detailed Molecular Dynamics (MD) analysis

We used atomistic molecular dynamics simulations to gain insights into the conformational properties of the imidazole ring attached to the purine ring of adenine residue nine in the DNA duplex. 3D structure of Imidazole labelled adenosine monophosphate (IMA) was generated in Gabedit [1]. Structure was capped at O3', and phosphate oxygen's using methyl groups before optimization. Using ORCA package [2,3], structure was optimized at restricted Hartree-Fock level of theory using 6-31g* basis set [4]. Using ambertools18 [6], AM1-BCC charges were calculated [5] and generalized amber force field [7] topology compatible with gromacs2018.2 [8-14] package was generated for IMA. Structure for duplex DNA was generated using make-na server (http://structure.usc.edu/make-na/server.html). IMA molecule was fitted onto DA9 residue, using only the purine ring atoms (only carbon and nitrogen) for the least square fitting, and coordinates of imidazole ring were transferred to pdb file creating the labelled DNA molecule (DIMA). DNA molecule was modelled using parmbsc1 forcefield [15]. DIMA was placed in a dodecahedron box with 1.0 nm distance between the box walls and DIMA. Simulation box was solvated with tip3p [16] water molecules and salt concentration was set 0.15M using Na^+ and Cl^- ions.

Simulation system was energy minimized using steepest descent algorithm until the largest force was smaller than 1000 kJ/mol/nm, followed by temperature equilibration to 300 K in 100ps using Berendsen thermostat [17] with a tau-t of 0.1 ps. In the succeeding step, pressure was equilibrated to 1 atm in 1 ns using Berendsen barostat [17] and temperature was regulated using velocity-rescaling thermostat [18] at 300 K. Using the equilibrated structure, three 100ns production run simulations were started in which temperature was regulated using velocity-rescaling thermostat and pressure with Parrinello-Rahman barostat [19] at 300 K and 1 atm using tau-t of 1ps and tau-p of 2ps. Structures were saved every 10 ps. All simulations were performed with a time step of 2fs, and all bonds were constrained using LINCS [20] and long range electrostatic interactions were calculated using PME [21]. A cut-off of 0.9 nm was applied for both short range electrostatic and Van der Waals interactions.

1. Gabedit—A graphical user interface for computational chemistry softwares. https://onlinelibrary.wiley.com/doi/full/10.1002/jcc.21600
2. Neese, F. (2012) The ORCA program system, Wiley Interdiscip. Rev.: Comput. Mol. Sci., 2, 73−78
3. Bykov, D.; Petrenko, T.; Izs´ak, R.; Kossmann, S.; Becker, U.; Valeev, E.; Neese, F. (2015) Efficient implementation of the analytic second derivatives of Hartree-Fock and

hybrid DFT energies: a detailed analysis of different approximations, Mol. Phys., 113, 1961.

4. W. J. Hehre, R. Ditchfield, and J. A. Pople. Self—Consistent Molecular Orbital Methods. XII. Further Extensions of Gaussian—Type Basis Sets for Use in Molecular Orbital Studies of Organic Molecules J. Chem. Phys. 56, 2257 (1972); https://doi.org/10.1063/1.1677527
5. Jakalian, A. , Bush, B. L., Jack, D. B. and Bayly, C. I. (2000), Fast, efficient generation of high-quality atomic charges. AM1-BCC model: I. Method. J. Comput. Chem., 21: 132-146. doi:10.1002/(SICI)1096-987X(20000130)21:2<132::AID-JCC5>3.0.CO;2-P
6. Wang, J., Wang, W., Kollman P. A.; Case, D. A. "Automatic atom type and bond type perception in molecular mechanical calculations". Journal of Molecular Graphics and Modelling , 25, 2006, 247260.
7. Wang, J., Wolf, R. M.; Caldwell, J. W.;Kollman, P. A.; Case, D. A. "Development and testing of a general AMBER force field". Journal of Computational Chemistry, 25, 2004, 1157-1174.
8. M. J. Abraham, T. Murtola, R. Schulz, S. Páll, J. C. Smith, B. Hess, E. Lindahl GROMACS: High performance molecular simulations through multi-level parallelism from laptops to supercomputer. SoftwareX 1 (2015) pp. 19-25
9. S. Páll, M. J. Abraham, C. Kutzner, B. Hess, E. Lindahl Tackling Exascale Software Challenges in Molecular Dynamics Simulations with GROMACS In S. Markidis & E. Laure (Eds.), Solving Software Challenges for Exascale 8759 (2015) pp. 3-27
10. S. Pronk, S. Páll, R. Schulz, P. Larsson, P. Bjelkmar, R. Apostolov, M. R. Shirts, J. C. Smith, P. M. Kasson, D. van der Spoel, B. Hess, and E. Lindahl GROMACS 4.5: a high-throughput and highly parallel open source molecular simulation toolkit Bioinformatics 29 (2013) pp. 845-54
11. B. Hess and C. Kutzner and D. van der Spoel and E. Lindahl GROMACS 4: Algorithms for highly efficient, load-balanced, and scalable molecular simulation J. Chem. Theory Comput. 4 (2008) pp. 435-447
12. D. van der Spoel, E. Lindahl, B. Hess, G. Groenhof, A. E. Mark and H. J. C. Berendsen GROMACS: Fast, Flexible and Free J. Comp. Chem. 26 (2005) pp. 1701-1719
13. E. Lindahl and B. Hess and D. van der Spoel GROMACS 3.0: A package for molecular simulation and trajectory analysis J. Mol. Mod. 7 (2001) pp. 306-317
14. H. J. C. Berendsen, D. van der Spoel and R. van Drunen GROMACS: A message-passing parallel molecular dynamics implementation Comp. Phys. Comm. 91 (1995) pp. 43-56

15. Parmbsc1: a refined force field for DNA simulations. Nature Methods volume 13, pages 55–58 (2016) https://www.nature.com/articles/nmeth.3658
16. Mahoney, Michael W, Jorgensen, William L. A five-site model for liquid water and the reproduction of the density anomaly by rigid, nonpolarizable potential functions. Journal of Chemical Physics; 112: 20; 8910-8922 https://aip.scitation.org/action/showCitFormats?type=show&doi=10.1063%2F1.481505
17. H. J. C. Berendsen, J. P. M. Postma, W. F. van Gunsteren, A. DiNola, and J. R. Haak Molecular dynamics with coupling to an external bath. J. Chem. Phys. 81, 3684 (1984); https://doi.org/10.1063/1.448118
18. Giovanni Bussi, Davide Donadio, and Michele Parrinello. Canonical sampling through velocity rescaling. J. Chem. Phys. 126, 014101 (2007); doi: 10.1063/1.2408420
19. Polymorphic transitions in single crystals: A new molecular dynamics method Journal of Applied Physics 52, 7182 (1981); https://doi.org/10.1063/1.328693
20. B. Hess and H. Bekker and H. J. C. Berendsen and J. G. E. M. Fraaije LINCS: A Linear Constraint Solver for molecular simulations J. Comp. Chem. 18 (1997) pp. 1463-1472
21. U. Essmann, L. Perera, M. L. Berkowitz, T. Darden, H. Lee and L. G. Pedersen A smooth particle mesh Ewald method J. Chem. Phys. 103 (1995) pp. 8577-8592
